# Modulation of Electronic Structure in Kraft Lignin‐Derived Mo Single‐Atom Catalysts for Optimized Electrochemical Oxygen Reduction

**DOI:** 10.1002/advs.202522273

**Published:** 2025-12-12

**Authors:** Junbeom Park, Jaemin Park, Jun Ho Seok, Ji Soo Byun, Cheoulwoo Oh, Eung‐Dab Kim, Young‐Jin Ko, Youngeun Kim, Gawon Sim, Min Jae Kim, Hyeon‐Seok Bang, Ho Seok Park, Chun‐Jae Yoo, Sang Uck Lee, Hyung‐Suk Oh, Kwang Ho Kim, Wooseok Yang

**Affiliations:** ^1^ School of Chemical Engineering Sungkyunkwan University (SKKU) Suwon 16419 Republic of Korea; ^2^ Clean Energy Research Center Korea Institute of Science and Technology (KIST) Seoul 02792 Republic of Korea; ^3^ School of Chemistry Monash University Clayton Victoria 3800 Australia; ^4^ Department of Chemical and Biomolecular Engineering Yonsei University Seoul 03722 Republic of Korea; ^5^ Department of Environmental and Energy Engineering Yonsei University Wonju 26493 Republic of Korea; ^6^ Department of Future Energy Engineering (DFEE) Sungkyunkwan University (SKKU) Suwon 16419 Republic of Korea; ^7^ School of Advanced Materials Science and Engineering Sungkyunkwan University (SKKU) Suwon 16419 Republic of Korea; ^8^ KIST‐SKKU Carbon‐Neutral Research Center Sungkyunkwan University (SKKU) Suwon 16419 Republic of Korea; ^9^ Department of Wood Science Faculty of Forestry University of British Columbia 2424 Main Mall Vancouver BC V6T 1Z4 Canada; ^10^ SKKU Institute of Energy Science and Technology (SIEST) Sungkyunkwan University Suwon 16419 Republic of Korea

**Keywords:** atomic dispersion, biomass, fuel cell, lignin‐derived catalyst, oxygen reduction reaction

## Abstract

The sluggish kinetics of the oxygen reduction reaction (ORR) remain a major bottleneck for energy conversion systems such as fuel cells and metal–air batteries. Here, the synthesis of molybdenum single‐atom catalysts (Mo SACs) derived from abundant and low‐cost Kraft lignin is reported. By tuning nitrogen incorporation during carbonization, agglomerated Mo carbide clusters are progressively converted into atomically dispersed Mo active centers anchored on N‐doped carbon. Extensive spectroscopic analyses confirm this structural evolution, while density functional theory calculations reveal that the optimized Mo coordination environment downshifts the d‐band center, enabling the balanced adsorption of oxygen intermediates and thereby improving the intrinsic ORR activity. Electrochemical measurements demonstrate enhanced half‐wave potential, near‐four‐electron transfer pathway, superior selectivity, and excellent durability, with ≈85% current retention over 50 h. Beyond performance, the use of minimally processed Kraft lignin underscores both the economic and environmental advantages of this approach, offering a scalable and sustainable pathway to practical ORR electrocatalysts.

## Graphical abstract

Biomass‐derived lignin is used as a sustainable precursor to synthesize Mo single atoms anchored on a nitrogen‐coordinated carbon framework. As the Mo structure transitions from MoC clusters to isolated single‐atom sites, oxygen evolution reaction (ORR) activity progressively improves. Density functional theory calculations reveal a corresponding downshift of the Mo d‐band center, optimizing oxygen intermediate binding and enhancing ORR kinetics.

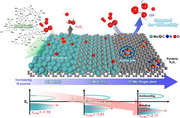

## 1 Introduction

The oxygen reduction reaction (ORR), a crucial process in energy conversion and storage devices (e.g., proton exchange membrane fuel cells and metal‐air batteries), is a major bottleneck owing to its sluggish kinetics and inefficient electron transfer.^[^ ^1^, ^2^, ^3^ ^]^ The complete reduction of O_2_ to H_2_O is essential for efficient power generation, making the 4e^−^ ORR pathway more favorable compared to the 2e^−^ ORR pathway to H_2_O_2_ generation.^[^ ^4^ ^]^ This pathway necessarily involves O–O bond cleavage; when this step is kinetically restricted, the reaction tends to proceed via the 2e^−^ pathway, yielding H_2_O_2_. While Pt‐based electrocatalysts accelerate the 4e^−^ pathway, their high cost and poor stability limit large‐scale commercial application. Among the various non‐precious metal catalysts, Mo‐based catalysts, which have strong binding energies that facilitate O–O bond cleavage and promote the formation of *O species, are potentially promising low‐cost alternatives for the efficient 4e^−^ ORR.^[^ ^5^ ^]^ However, according to the Sabatier principle, excessive binding interactions hinder the desorption of O and OH intermediates, thus limiting the efficiency of the 4e^−^ ORR.^[^ ^6^ ^]^ The electronic structure, particularly the d‐band center (*ε_d_*), can determine the bond strength between the catalyst and intermediates. As adsorbate p/s states hybridize with d states of the transition metals, the position of ε_d_ relative to the Fermi level (E*_f_*) dictates antibonding‐state occupancy; a deeper ε_d_ (farther below E*_f_*) increases antibonding filling and weakens adsorption, whereas an ε_d_ closer to E*_f_* reduces antibonding occupancy and strengthens the bond.^[^ ^7^, ^8^ ^]^ Therefore, the intermediate adsorption/desorption can be optimized by modulating the *ε_d_* of Mo‐based catalysts and improving the intrinsic catalytic activity toward ORR.

Recent studies have demonstrated that reducing the size of metal particles to the single‐atom level can significantly enhance the catalytic performance by maximizing atomic utilization and leveraging the unique size quantum effect.^[^ ^9^, ^10^ ^]^ Single‐atom catalysts (SACs) offer distinct advantages that enable the rational design of materials at the atomic scale.^[^ ^11^ ^]^ The coordination environment of SACs, including the interactions between the metal center and surrounding atoms, plays a pivotal role in determining their catalytic efficiency. For example, the catalytic activity of transition metal and nitrogen co‐doped carbon‐based (M–N_x_–C) SACs is contingent upon key factors such as atomic coordination, the presence and type of heteroatoms, and their interactions within the M–N_x_–C moieties.^[^ ^12^, ^13^ ^]^ In this regard, well‐defined SACs can serve as ideal models, enabling the enhancement of intrinsic catalytic properties through atomic‐level insights into active centers and a deeper understanding of catalytic reaction mechanisms.

Conventional SAC synthesis often relies on coordination ligands to stabilize isolated metal atoms and prevent agglomeration.^[^ ^14^ ^]^ However, these ligands are frequently toxic, expensive, and environmentally unsustainable. Lignin—a 3D amorphous phenolic biopolymer abundant in wood and non‐wood biomass—presents a greener alternative.^[^ ^15^ ^]^ Rich in oxygen‐containing functionalities, lignin can readily chelate transition‐metal ions through phenolic and carboxylate groups, forming metal–lignin supramolecular complexes that promote atomic dispersion.^[^ ^16^ ^]^ Among lignin sources, Kraft lignin is the least refined, most heterogeneous, and most abundant byproduct of the pulp and paper industry. It is highly accessible and cost‐effective, yet remains vastly underutilized: of the ≈50 million tons produced annually, the majority is simply incinerated or discarded.^[^ ^17^, ^18^, ^19^ ^]^ Its variability in sulfur/sodium content and molecular weight poses challenges for reproducible functionalization,^[^ ^20^ ^]^ but successful valorization of Kraft lignin as a catalyst support could transform an industrial “waste” stream into a valuable precursor for sustainable energy applications.

Here, we present a Kraft lignin–derived synthesis strategy that drives the controlled transition from Mo carbide clusters to atomically dispersed Mo SACs. We identify nitrogen incorporation as a key parameter governing this structural evolution and demonstrate tunable active‐site density through nitrogen control. Furthermore, our results not only confirm the successful phase transition but also highlight the concurrent modulation of electronic configuration. Density functional theory (DFT) calculations revealed a tailored d‐band structure that optimizes intermediate binding and improves intrinsic ORR activity. The optimized Mo SAC achieved a half‐wave potential of 0.88 V_RHE_ and an electron‐transfer number (n) of 3.82, confirming the predominance of the 4e^−^ pathway. These enhancements arise from the synergy of increased active‐site density via atomic dispersion and intrinsic improvements in electronic structure. Importantly, using Kraft lignin as a carbon precursor reduces costs by more than 90% relative to conventional carbon sources, while simultaneously advancing sustainability. This work therefore demonstrates not only a practical route to high‐performance Mo‐based SACs, but also an economically and environmentally viable strategy for ORR electrocatalyst design, with broad implications for scalable clean‐energy technologies.

## 2 Results and Discussion

**Figure 1:**
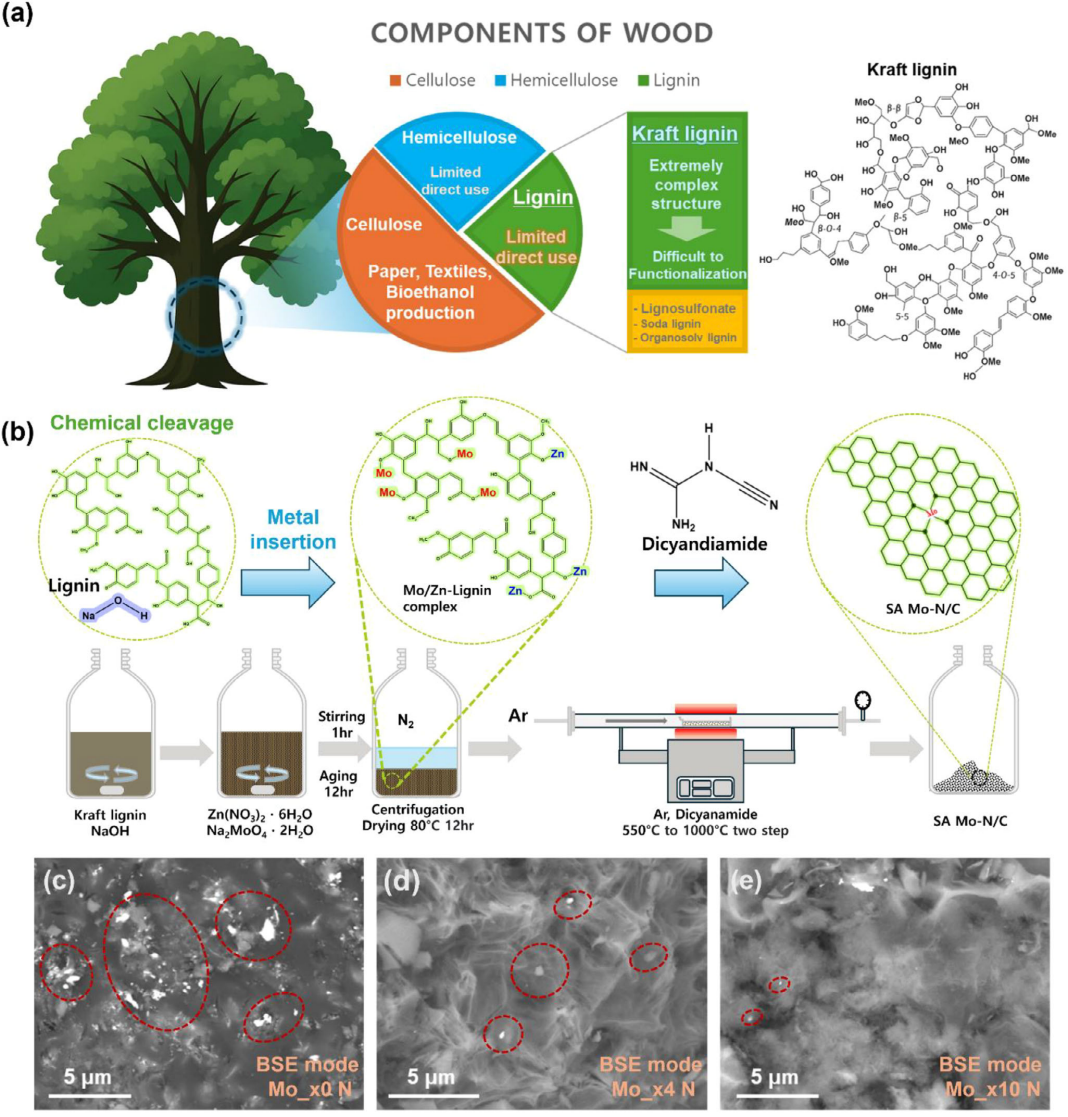
a) Schematic of wood composition (cellulose, hemicellulose, and lignin) and the limited direct use of lignin. b) Proposed synthetic route for a Kraft‐lignin‐derived single‐atom Mo–N/C catalyst. c) SEM images (back scattered‐electron mode) of products obtained with increasing Mo/N precursor ratios: (c) Mo ×0 N, d) Mo ×4 N, and e) Mo ×10 N. Red dashed circles mark agglomerated Mo‐containing particles.

Among the three major components of lignocellulosic biomass, Kraft lignin represents a substantial and underutilized fraction. Its effective valorization would provide both economic and environmental benefits (**Figure** **1**a).^[^ ^21^ ^]^ Here, we developed a Mo SAC catalyst from Kraft lignin through chemical cleavage–assisted metal coordination, followed by pyrolysis. The precursor was obtained by co‐mixing Kraft lignin with Mo and Zn salts, then treated with varying amounts of dicyandiamide (DCD) as a nitrogen source (Figure 1b; detailed procedures in the Experimental Section). FTIR analysis (Figure S1, Supporting Information) revealed a redshift of the –OH stretching band (≈3500 cm^−1^) upon metal incorporation, consistent with coordination of lignin hydroxyl groups to metal ions. During pyrolysis, Zn(NO_3_)_2_ volatilizes, and subsequent carbonization and post‐treatment remove both zinc and sulfur from the catalyst. This process increases the spacing between Mo species within the matrix and facilitates nitrogen doping of the carbon framework.^[^ ^22^ ^]^ Catalysts were synthesized using DCD as the nitrogen source at different weight ratios of 0 times (no DCD), 4 times, and 10 times the Mo amount to tune the degree of nitrogen incorporation. The resulting samples are denoted as Mo_x0 N, Mo_x4 N, and Mo_x10 N, respectively.

Morphological analyses of Mo_x0 N, Mo_x4 N, and Mo_x10 N were performed using scanning electron microscopy (SEM) (Figure S2, Supporting Information). In the secondary electron mode, all three samples exhibited similar irregular carbon fringes without significant distinctions. However, as shown in Figure 1c–e, backscattered electron (BSE) mode imaging, which is sensitive to atomic number contrast, revealed bright regions corresponding to the Mo species owing to their high atomic number. In the Mo_x0 N sample, large and bulk‐like bright Mo agglomerates were observed. As the nitrogen content increased, a progressive reduction in both the size and abundance of the Mo agglomerates resulted in the negligible detection of Mo_x10 N.

**Figure 2:**
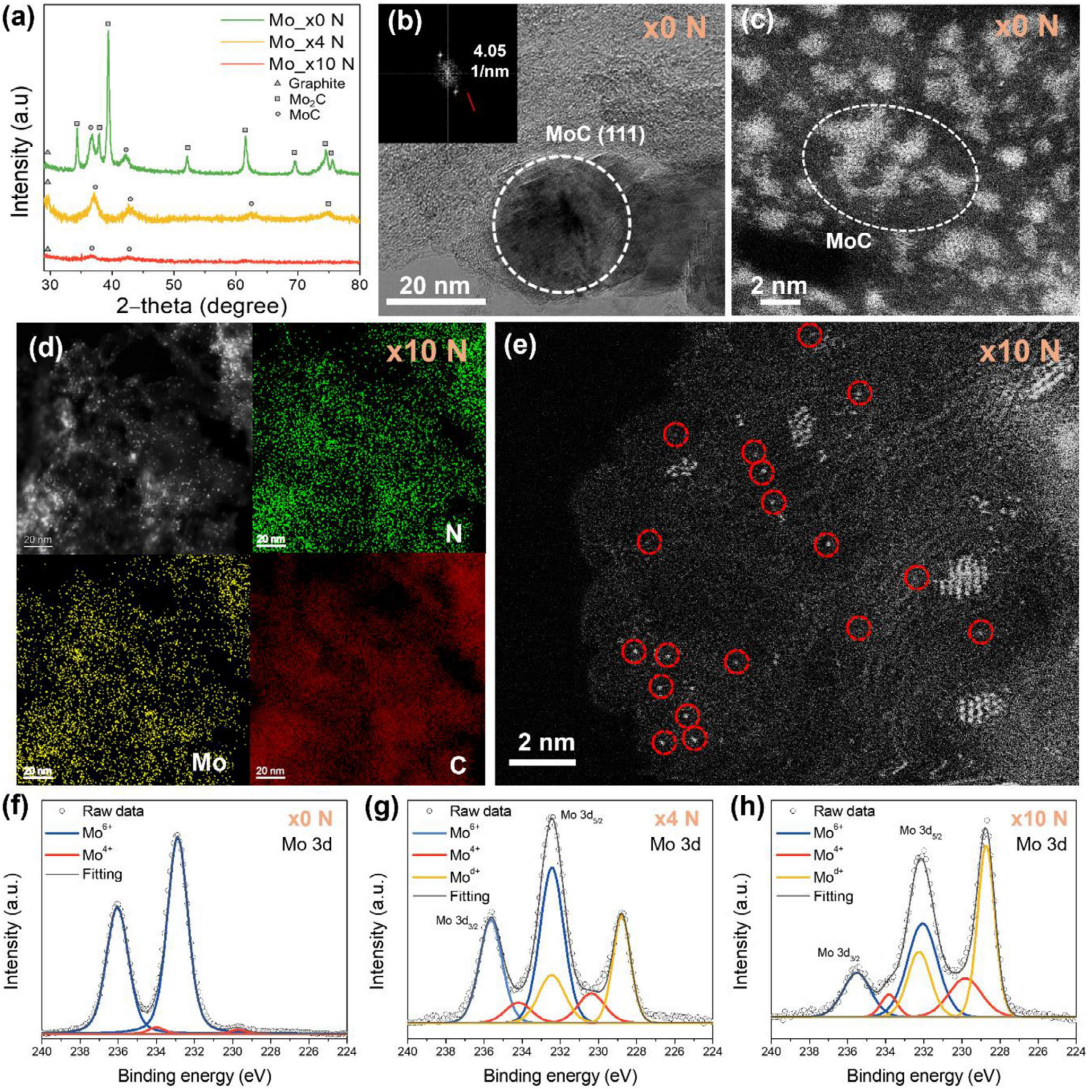
a) X‐ray diffraction patterns of Mo_x0 N, Mo_x4 N, and Mo_x10 N samples. b) Low‐magnification TEM image of Mo x0 N showing a MoC particle (dashed circle) with a (111) lattice fringe spacing of 0.24 nm (FFT inset). c) HRTEM image of Mo_x0 N. The dashed oval highlights a MoC nanoparticle. d) highlights a MoC nanoparticle. (d) STEM–EDS elemental maps for Mo x10 N confirm a homogeneous distribution of C, N, and Mo. e) HAADF‐STEM image of Mo x10 N; red circles mark isolated bright spots corresponding to single Mo atoms anchored on the carbon matrix. High‐resolution Mo 3d XPS spectra and peak deconvolution for f) Mo x0 N, g) Mo x4 N, and h) Mo x10 N.

A comprehensive characterization was conducted using X‐ray diffraction (XRD), transmission electron microscopy (TEM), and high‐angle annular dark‐field scanning transmission electron microscopy (HAADF‐STEM) to further investigate the structural evolution and atomic dispersion of the Mo species. As shown in **Figure** **2**a, the XRD pattern of Mo_x0 N displays distinct peaks corresponding to MoC and Mo_2_C, indicating the formation of crystalline molybdenum carbides. These findings confirm that the bright agglomerated domains observed in Figure 1c originate from the molybdenum carbides. However, these characteristic peaks were significantly weakened in Mo_x4 N and nearly disappeared in Mo_x10 N, suggesting progressive suppression of MoC crystallization with increasing nitrogen content. This trend is further supported by the TEM images shown in Figure 2b–e. Mo_x0 N displays distinct crystalline MoC and Mo_2_C domains (Figures S3 and S4, Supporting Information), whereas Mo_x4 N and Mo_x10 N exhibit progressive transitions toward more amorphous and finely dispersed structures. Energy‐dispersive X‐ray spectroscopy (EDS) mapping confirms the uniform distribution of Mo, N, and C throughout the Mo_x10 N catalyst, with no observable Mo agglomerates larger than 5 nm within the carbon matrix (Figure 2d). Furthermore, the HAADF‐STEM image of Mo_x10 N reveals well‐dispersed bright spots (highlighted by red circles), indicating isolated Mo atoms anchored on the N‐doped carbon framework (Figure 2e). Additional low‐magnification TEM and HAADF‐STEM images of Mo_x4 N and Mo_x10 N are provided in Figures S5 and S6 (Supporting Information), further highlighting the distinct size and abundance of the reduced Mo agglomerates.

High‐resolution X‐ray photoelectron spectroscopy (XPS) analysis was used to investigate the surface chemical states. The deconvolution of the C 1s spectra in Figure S7 (Supporting Information) shows that the increased intensity of C–N bonding (≈286 eV) with higher nitrogen content indicates effective nitrogen doping.^[^ ^23^ ^]^ In addition, deconvolution of the Mo 3d spectra reveals the presence of three oxidation states: Mo⁴⁺, Mo⁶⁺, and Mo^δ⁺^ (0 < δ < 4) in Figure 2f–h.^[^ ^24^ ^]^ In the Mo_x0 N sample, the dominant Mo⁴⁺ and Mo⁶⁺ species are primarily attributed to MoO_2_ and MoO_3_, respectively, which are commonly observed as carbides that are exposed to air (Figure 2f).^[^ ^25^ ^]^ Notably, following nitrogen incorporation, a new peak emerges at 228.5 eV in Figure 2g, assigned to Mo^δ⁺^ species,^[^ ^24^ ^]^ and this contribution increases significantly in the Mo_x10 N sample. The systematic increase in the Mo^δ⁺^ signal with higher nitrogen content, in conjunction with the confirmed nitrogen incorporation, provides compelling evidence that enhanced Mo^δ⁺^ signal species originate from the formation of Mo–N bonds at the surface.^[^ ^26^ ^]^ Moreover, deconvolution of the N 1s spectra reveals increased Mo–N bonding in Mo_x4 N and Mo_x10 N compared to Mo_x0 N in Figure S8 (Supporting Information), further confirming that the enhanced Mo^𝛿+^ signal originates from the formation of Mo–N bonds. It should be noted here that the Mo 3d peaks shift to lower binding energies with increasing nitrogen content (Figure 2f–h), whereas the pyridinic‐ and pyrrolic‐N components in the N 1s spectrum move to higher binding energies (Figure S8, Supporting Information).^[^ ^27^ ^]^ Pyridinic and pyrrolic nitrogen species are well‐known π‐electron donors and common coordination sites for Mo centers.^[^ ^28^ ^]^ These opposite shifts, therefore, indicate electron transfer from N ligands to Mo, resulting in a modified local electronic structure around the Mo atoms.

**Figure 3:**
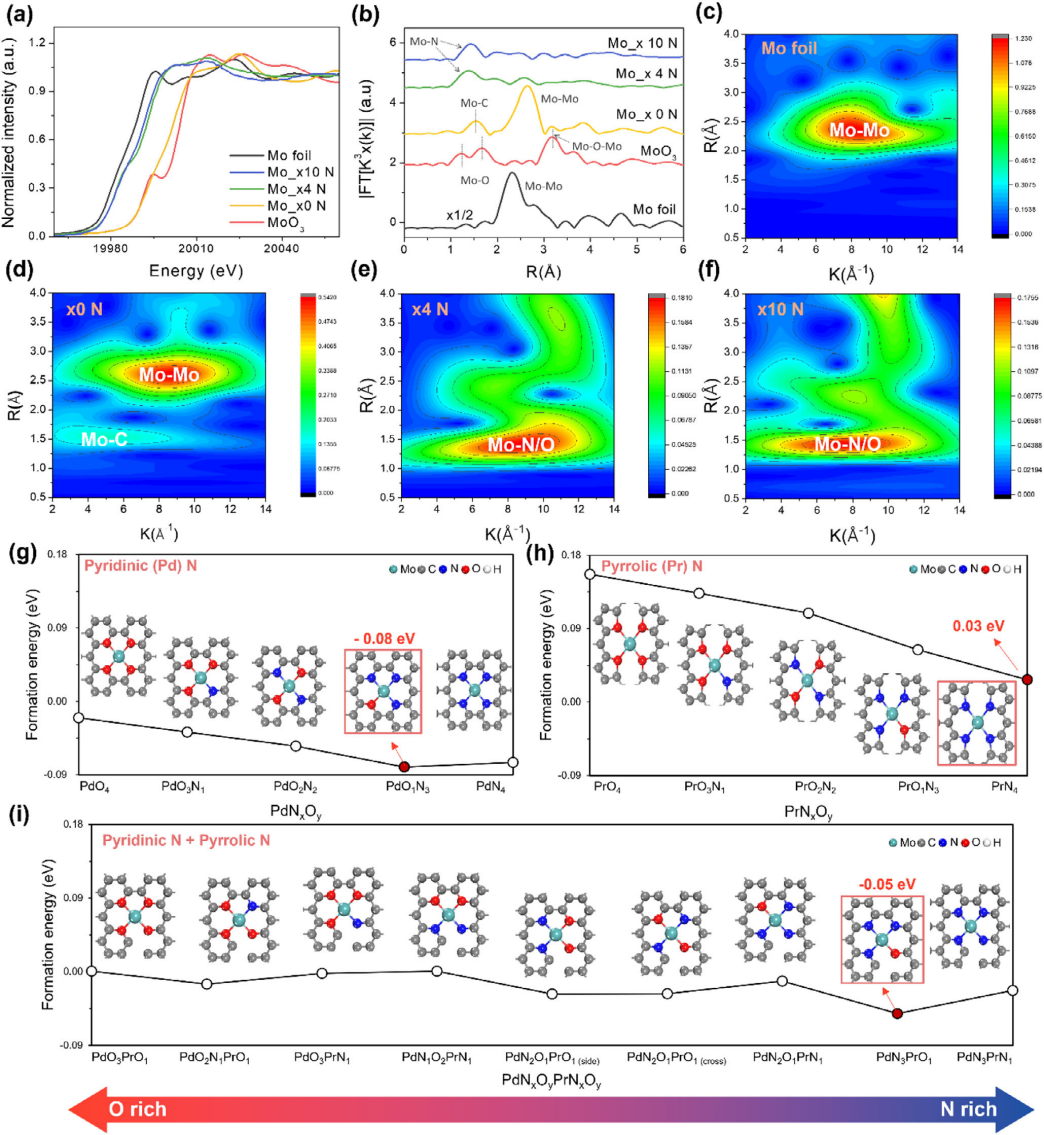
a) Normalized Mo K‐edge XANES spectra and b) k^3^‐weighted Fourier‐transformed EXAFS (2 ≤ k ≤ 12 Å^−1^) of Mo x0 N, Mo x4 N, and Mo x10 N compared with Mo foil and MoO_3_ references. Wavelet‐transform (WT) contour plots of EXAFS signals for c) Mo foil, d) Mo x0 N, e) Mo x4 N, and f) Mo x10 N. The high‐k Mo–Mo maximum (≈ 8–12 Å^−1^; R ≈ 2.7 Å). Calculated formation energies (*E_for_*) of Mo in g) pyridinic (Pd) N/O, h) pyrrolic (Pr) N/O, and i) mixed pyridinic/pyrrolic N/O coordination environments. The most thermodynamically stable configuration in each group is highlighted with a red box.

X‐ray absorption near‐edge structure (XANES) and extended X‐ray absorption fine structure (EXAFS) spectroscopy were employed to investigate the local coordination, chemical composition, and surroundings of Mo elements at the atomic level. As shown in **Figure** **3**a, the Mo K‐edge XANES curves reveal that the absorption edges of Mo_x0 N, Mo_x4 N, and Mo_x10 N lie between those of the Mo foil and MoO_3_, implying that the Mo valences in these catalysts are located between those of the two references (i.e., the 0 and +6 valence states, respectively). In addition, a distinct shift to lower energy values was observed with increasing nitrogen content. This behavior parallels the Mo 3d XPS results, which are attributed to electron donation from the π‐electron–rich pyridinic and pyrrolic nitrogen ligands, which increase the d‐electron density at the Mo and thus lower their valence state. The local coordination environment of Mo was probed using Mo K‐edge EXAFS spectroscopy. As shown in Figure 3b, the R‐space spectra of Mo_x4 N and Mo_x10 N showed a prominent peak near 1.4 Å, assigned to the Mo–N scattering path. In contrast, the metallic Mo foil displays a Mo–Mo peak at ≈2.4 Å. The absence of any Mo–Mo features in Mo_x4 N and Mo_x10 N, therefore, indicates that, with nitrogen incorporation, Mo is predominantly atomically dispersed, whereas Mo_x0 N still contains Mo–Mo coordinated domains.

The atomic dispersion of Mo in Mo_x10 ​N was further confirmed by EXAFS wavelet transform (WT‐EXAFS) analysis (Figure 3c–f). The metallic Mo reference (Figure 3c) exhibits a single, high‐intensity lobe centered at k ≈ 8.5 Å^−1^ and R ≈ 2.4 Å, which is unequivocally assigned to Mo–Mo coordination. The undoped composite Mo_x0 N (Figure 3d) retains this Mo–Mo signature, confirming that, in the absence of nitrogen, Mo persists as clusters or carbide‐like domains. It should be noted that the high‐intensity lobe centered at k ≈ 5 Å^−1^ and R ≈ 1.5 Å in the Mo_x0 N spectrum corresponds to Mo‐C coordination, which distinguished from the WT‐EXAFS spectra of MoO_3_ (Figure S9, Supporting Information), Mo_x4 N, and Mo_x10 N (Figure 3e,f, respectively), where Mo–O/N coordination is observed in Mo_x4 N and Mo_x10 N,^[^ ^29^, ^30^, ^31^ ^]^ and Mo‐O coordination is present in MoO_3_. The Mo–C coordination exhibits a higher intensity at lower k and higher R values compared to Mo–N and Mo–O, owing to the weaker Mo‐C bond strength and longer bond length, which results in lower scattering wave vectors. In the Mo_x4 N (Figure 3e), the Mo–Mo lobe is completely extinguished and supplanted by an isolated intensity maximum at k ≈ 5.5 Å^−1^ and R ≈ 1.4 Å, diagnostic of first‐shell Mo–N/O scattering.^[^ ^29^, ^30^, ^31^ ^]^ On further enrichment to Mo_x10 N (Figure 3f), this Mo–N/O feature both intensifies and migrates to lower values (k ≈ 5.2 Å^−1^ and R ≈ 1.3 Å).

The concurrent reduction in k and R is consistent with a shortened Mo–N bond length and the increasing dominance of light first‐shell scatterers, indicating tighter, more highly coordinated Mo–N_x_ geometries of Mo_x10 N. Taken together, these observations reveal a progressive transition from Mo clusters or carbide to atomically dispersed Mo at high nitrogen loadings, in full accord with the XRD and AC‐HAADF‐STEM results. These results demonstrate the successful development of a controllable Kraft‐lignin‐derived synthesis strategy that enables the modulation of MoC formation to atomically dispersed Mo species, ultimately establishing a methodology that facilitates the concurrent control of the phase transition and electronic configuration. Although minor residual domains may be observed in the TEM images due to the intrinsic heterogeneity of the minimally processed Kraft lignin used as a biomass precursor, the structure is unequivocally dominated by Mo single atoms, as further confirmed by the relatively bulk‐averaged XAS analysis.

Nitrogen incorporation during the synthesis can lead to diverse Mo coordination environments, including pyridinic (Pd), pyrrolic (Pr), and partially oxygen‐substituted N/O mixed sites; however, the predominant configuration cannot be unambiguously determined from experimental data alone. To elucidate the atomic arrangement more precisely and rationalize the observed results, DFT calculations were systematically performed on the models of the Mo clusters, MoC, and Mo SACs. Based on the XPS results (Figure S8, Supporting Information), three representative coordination models were constructed for the Mo SAC: Mo@PdN_4_ (pyridinic N_4_), Mo@PrN_4_ (pyrrolic N_4_), and Mo@PdN_3_PrN_1_ (mixed pyridinic N_3_/pyrrolic N_1_), as depicted in Figure S10a–c (Supporting Information). For Mo_x0 N, the Mo(111) and MoC(111) surfaces were included as reference structures (Figure S10d,e, Supporting Information). Details of the model construction are provided in the computational details section. All the possible coordination environments of the Mo SACs were classified into three groups. Their thermodynamic stability was assessed by calculating the formation energies (*E_for_*) of i) PdN*_x_* O*_y_*, ii) PrN*_x_* O*_y_*, and iii) PdN*_x_* O*_y_*/PrN*_x_* O*_y_* mixed structures (*x* + *y* = 4). All models were thermodynamically stable, and their relative stabilities were dependent on the coordination environment (Figure 3g–i). Although the calculated formation energy of PrN*_x_* O*_y_* is positive (0.03–0.16 eV), it lies within the commonly accepted ≈0.3 eV metastability window,^[^ ^32^ ^]^ indicating that such structures should remain synthetically accessible under experimental conditions. Among the three configurations, PdN*_x_* O*_y_* ​ was the most stable, exhibiting the lowest formation energy, which was in agreement with the XPS results showing that pyridinic N was the dominant feature (Figure S8, Supporting Information). The details of the formation energy calculations are provided in Note S1 (Supporting Information). The formation energy analysis revealed that Mo@PdN_3_O_1_, Mo@PrN_4_, and Mo@PdN_3_PrO_1_ were the most stable within their respective groups, underscoring the energetic preference for partially oxygen‐substituted N/O mixed configurations. These results indicate that the Mo SACs are not confined to a single Mo‐N_4_ configuration, but rather exist as a combination of pyridinic, pyrrolic, and N/O mixed coordination environments.

**Figure 4:**
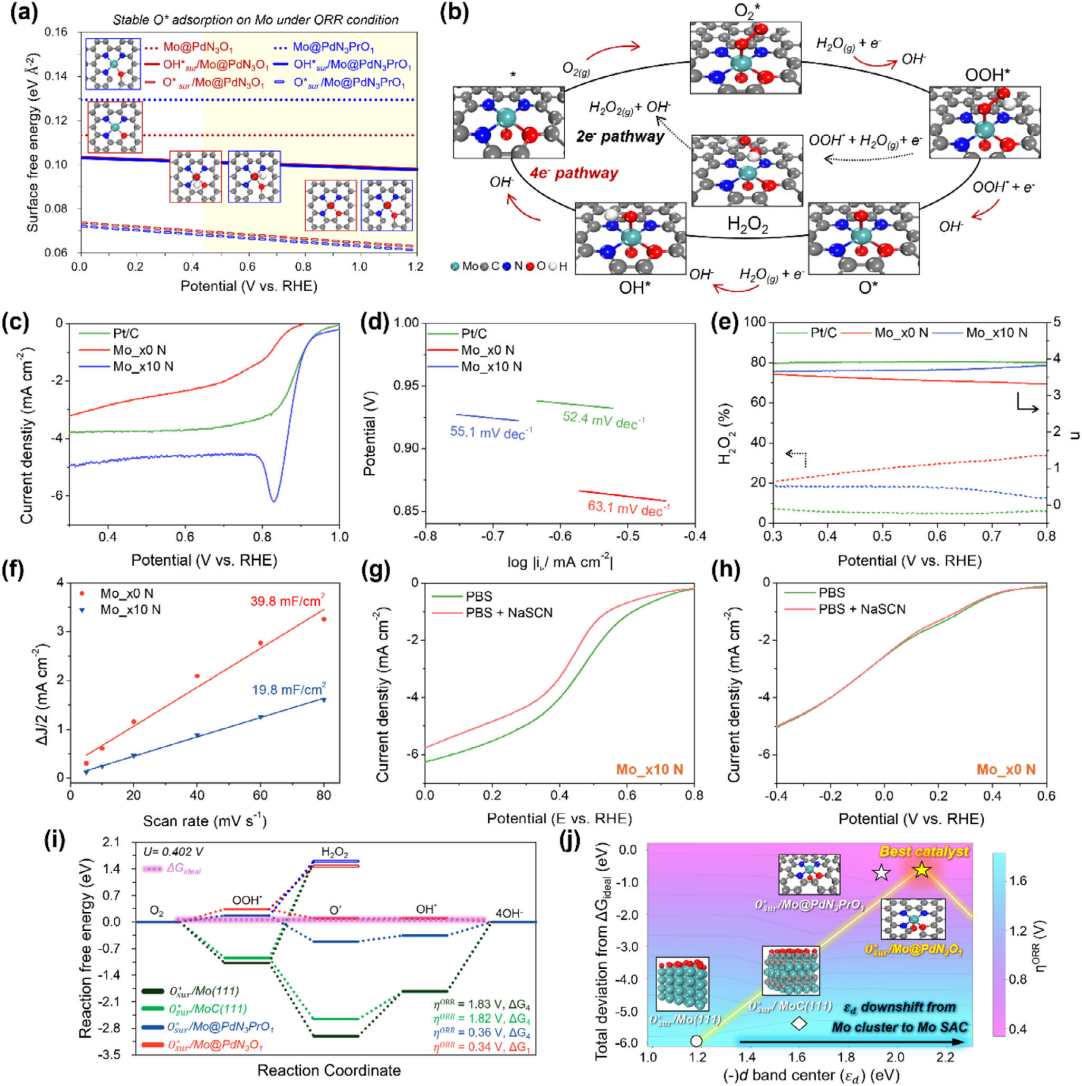
a) Calculated surface free energies as a function of the applied potential for Mo@PdN_3_PrO_1_ and Mo@PdN_3_O_1_ in a clean surface, $O_{sur}^{*}$, and $OH_{sur}^{*}$. The yellow shaded region denotes the ORR‐relevant potential range. b) Schematic ORR mechanism in an alkaline medium. The black arrows represent the 2e^−^ pathway, while red arrows represent the 4e^−^ pathway. c) ORR polarization curves for commercial Pt/C, Mo x0 N, and Mo x10 N, and d) corresponding Tafel plots. e) n and generated H_2_O_2_ calculated from RRDE measurements. f) C_dl_ extracted from ΔJ/2 versus scan‐rate plots. ORR polarization curves of g) Mo_x10 N and h) Mo_x0 N in PBS electrolyte before and after addition of NaSCN. i) FED for ORR on $O_{sur}^{*}$/Mo(111), $O_{sur}^{*}$/MoC(111), $O_{sur}^{*}$/Mo@PdN_3_PrO_1_, and $O_{sur}^{*}$/Mo@PdN_3_O_1_. The violet dotted line indicated ideal free energy. j) Contour plot showing the relationship between the Δ*G_dev_* and *ε_d_* of the Mo‐based catalysts.

Prior to evaluating electrocatalytic performance, it is well established that Mo‐based catalysts undergo surface reconstruction under electrochemical conditions that critically influence their final electrocatalytic behavior.^[^ ^33^, ^34^, ^35^ ^]^ Recent experimental and theoretical studies have indicated that oxygen species ($O_{sur}^{*}\;$ and $OH_{sur}^{*}$) can passivate and stabilize Mo SAC active sites in a potential‐dependent manner under alkaline ORR conditions.^[^ ^36^ ^]^ Models including Mo(111), MoC(111), Mo@PdN_3_O_1_, and Mo@PdN_3_PrO_1_ with oxygen species were constructed to simulate oxygen species passivation. The crystal lattices were cleaved along the representative low‐index (111) facet, based on the HRTEM observations in Figure 2b. The thermodynamic stabilities of all the models were evaluated by comparing the surface free energies at ORR‐relevant potentials (details of the surface free energy calculations are provided in Note S2, Supporting Information).^[^ ^37^, ^38^, ^39^ ^]^ As illustrated in **Figures** **4**a and S11 (Supporting Information), the $O_{sur}^{*}$ configurations consistently exhibited the lowest surface energy (e.g., the highest thermodynamic stability) across all models under ORR‐relevant potentials. These findings indicate that $O_{sur}^{*}\;$ passivation of Mo defines the active species, with the $O_{sur}^{*}$/Mo‐based catalyst serving as the functional center for the ORR. It should be noted here that the oxygen source of O^*^ passivation originates from the adsorbed oxygen species in the electrolyte, induced by the presence of OH^−^. Subsequently, we systematically investigated the ORR, which proceeds through the 2e^−^ pathway (H_2_O_2_ formation) and the 4e^−^ pathway (OH^−^ formation) pathways.^[^ ^40^ ^]^ As shown in Figure 4b, the reaction followed an identical mechanism before and after the $O_{sur\;}^{*}$ passivation process.

The electrocatalytic performance of the Kraft lignin‐derived catalysts for ORR was evaluated using the rotating ring‐disk electrode (RRDE) technique in a 1.0 m KOH electrolyte. Cyclic voltammograms (CV) were obtained (Figure S12, Supporting Information) in electrolytes purged with N_2_ or O_2_ for ≥20 min, with continuous bubbling. Under N_2_, no potential peak was observed, whereas under O_2_, a pronounced cathodic peak appeared at 0.80 V_RHE_​ for Mo_x10N, the first indication of its high ORR activity and facile oxygen reduction.^[^ ^41^ ^]^ This trend is corroborated by the RRDE polarization curves (Figure 4c), where the commercial Pt/C was used for comparison. The half‐wave potentials (E_1/2_​) and diffusion‐limited current densities (J_lim_​) summarized in Figure S13 (Supporting Information) clearly demonstrate the performance enhancement from Mo_x0 ​N to Mo_x10 ​N. Specifically, Mo_x10 ​N (J_lim_​= –5 mA cm^−2^; E_1/2_​= 0.88 V_RHE_​) exhibits substantially higher activity than Mo_x0 ​N (J_lim_​= –3.2 mA cm^−2^; E_1/2_​= 0.75 V_RHE_​) and even surpasses the J_lim_​ of Pt/C (J_lim_​= –3.8 mA cm^−2^; E_1/2_​= 0.92 V_RHE_​). Kinetic analysis further supports enhanced catalytic activity in that the Tafel slope of Mo_x10 ​N is 55.1 mV dec^−1^, closely matching that of Pt/C (52.4 mV dec^−1^), evidencing rapid ORR kinetics on Mo_x10 ​N (Figure 4d). The selectivity derived from the ring current (Figure 4e) showed that the H_2_O_2_ yield for Mo_x10 ​N was far lower than that for Mo_x0 ​N, highlighting the strong preference for the four‐electron pathway for H_2_​O. The corresponding n for Mo_x10 ​N is maintained at 3.65–3.8 within 0.6–0.8 V_RHE_​, consistent with mainly a 4e^−^ ORR pathway. Furthermore, we conducted RRDE LSV measurements on multiple independently prepared samples to ensure reproducibility. The curves obtained showed consistent electrochemical behavior with the representative dataset, indicating good sample reproducibility (Figure S14, Supporting Information).

The electrochemical surface area (ECSA), estimated from the double‐layer capacitance (C_dl_​, Figure S15, Supporting Information), was used to probe the surface characteristics of the catalysts (Figure 4f). Mo_x0 ​N exhibited a higher C_dl_​ value (26.2 mF cm^−2^) than Mo_x10 ​N (19.8 mF cm^−2^), reflecting its larger electrochemical capacitance. Given that C_dl_ is measured in the non‐Faradaic potential region and is strongly influenced by the surface morphology, this result indicates, in a restricted sense, the relatively rougher surface morphology of Mo_x0 ​N observed in the SEM and HRTEM images. The specific activity (J_s_​, current density normalized to ECSA) was analyzed as a function of overpotential (Figure S16, Supporting Information) to compare the intrinsic catalytic activity. Mo_x10​ N requires a lower potential than Mo_x0 ​N to attain the same current density, demonstrating its superior intrinsic ORR activity. To further elucidate the role of single Mo atoms in generating ORR‐active sites responsible for the enhanced intrinsic ORR activity, the ORR performances of Mo_x0 ​N and Mo_x10 ​N were examined in 1x phosphate‐buffered saline (PBS) containing 5 mm NaSCN. In this system, SCN^−^ anions selectively coordinate with Mo centers and deactivate Mo‐based sites. As shown in Figures 4g,h, Mo_x10 ​N displayed a notable decrease in onset potential and J_lim_​ upon the introduction of SCN^−^, whereas Mo_x0 ​N remained unaffected. The observed performance difference in the presence and absence of SCN^−^ suggests that the Mo single‐atom sites in Mo_x10 N serve as the active sites for ORR. Accordingly, the enhanced intrinsic activity can be attributed to the restructuring of the active sites into single‐atom sites, whereas the overall improvement in the ORR performance arises from the synergistic effect of the increased Mo single‐atom site density and enhanced intrinsic activity.

Given that Mo persists as clusters or carbide‐like domains in Mo_x0 N but exists as atomically dispersed single atoms in Mo_x10 N, free energy diagrams (FEDs) for ORR ^[^ ^42^, ^43^ ^]^ were calculated for Mo, MoC, and Mo SACs along both the 2e^−^ and 4e^−^ pathways to elucidate the fundamental origin of the enhanced intrinsic ORR activity observed in Mo_x10 N (Figure 4i). To investigate catalytic reactions on specific surface structures, we generally employ the thermodynamic stability of the intermediates as the main descriptor, which determines the catalytic performance. A detailed evaluation of the ORR pathway and calculation of ORR activity in alkaline environments are provided in Note S3 (Supporting Information). Prior to comparing Mo‐based catalysts, we note that $O_{sur}^{*}$​–passivated sites represent the thermodynamically preferred active configuration under ORR‐relevant potentials (Figure 4a); accordingly, all FEDs were computed for $O_{sur}^{*}$/Mo‐based models. Upon $O_{sur}^{*}$​ passivation, the η^ORR^​ decreases markedly relative to the pristine Mo surface (i.e., in the absence of $O_{sur}^{*}$∗​; Figures S17–S19, Supporting Information). In addition, a significantly high OOH^*^ → H_2_O_2_ energy barrier was exhibited across all structures, effectively suppressing H_2_O_2_ formation (i.e., 2e^−^ pathway) and consistently promoting selective H_2_O production (i.e., 4e^−^ pathway), consistent with the strong Mo–intermediate binding that facilitates O–O bond cleavage.

The calculated η^ORR^​ values for $O_{sur}^{*}$​/Mo(111) and $O_{sur}^{*}$/MoC(111) are 1.83 and 1.82 V, respectively, which remain relatively high, with the final reduction step ($\Delta G_{4}^{ORR}$: OH^*^ → OH^−^) serving as the potential‐determining step (PDS). By comparison, $O_{sur}^{*}$/Mo SACs (e.g., i) $O_{sur}^{*}$ /Mo@PdN*_x_* O*_y_*, ii) $O_{sur}^{*}$ /Mo@PrN*_x_* O*_y_*, and iii) $O_{sur}^{*}$ /Mo@PdN*_x_* O*_y_*/PrN*_x_* O*_y_* mixed structures) showed significantly reduced η^ORR^ values. For $O_{sur}^{*}$/Mo@PdN_3_PrO_1_, the PDS remained at $\Delta G_{4}^{ORR}$, but the η^ORR^ dropped sharply to 0.36 V. Remarkably, $O_{sur}^{*}$/Mo@PdN_3_O_1_ achieved optimally moderated OH binding, shifting the PDS from $\Delta G_{4}^{ORR}$ to the first reduction step ($\Delta G_{1}^{ORR}$: O_2_ → OOH^*^) and reducing η^ORR^ further to 0.34 V (Table S1, Supporting Information). At the ideal ORR potential under alkaline conditions (U = 0.402 V), an ideal catalyst would exhibit zero chemical potential for all intermediates, with no associated reaction energy barriers, as indicated by the violet dotted line in Figure 4i. Accordingly, the nonzero reaction free energies of the intermediates reflect deviations from this ideal behavior, giving rise to overpotentials. To quantify such deviations, the non‐ideality of a catalyst is evaluated as the sum of the absolute values of the reaction free energies, defined as Δ*G_dev_* =  ∑|Δ*G_i_* |, providing a metric for assessing catalytic performance. $O_{sur}^{*}/\mathrm{Mo}$(111) and $O_{sur}^{*}/\mathrm{MoC}$(111) exhibit substantial deviations from the ideal zero, with Δ*G_dev_* values of 5.91 and 5.34 eV, respectively, reflecting limitations due to excessively strong adsorption. In contrast, $O_{sur}^{*}/\mathrm{Mo}$@PdN_3_PrO_1_ and $O_{sur}^{*}/\mathrm{Mo}$@PdN_3_O_1_ show much lower Δ*G_dev_* values of 0.70 and 0.54 eV, indicating near‐ideal catalytic behavior. The position of ε_d_ relative to E*_f_* dictates antibonding‐state occupancy and thereby governs adsorption strength; accordingly, the calculated volcano plot in Figure 4j illustrates the correlation among ∆G_dev_, η^ORR^, and adsorption strength.^[^ ^44^, ^45^ ^]^ It further confirms that a smaller Δ*G_dev_* correlates with balanced adsorption strength, which consequently leads to a lower η^ORR^. These results clearly demonstrate that the transition from Mo_x0 N (i.e., Mo cluster and MoC) to Mo_x10 N (i.e., Mo SACs) results in enhanced intrinsic ORR activity through an optimal binding strength, as described by the Sabatier principle.

**Figure 5:**
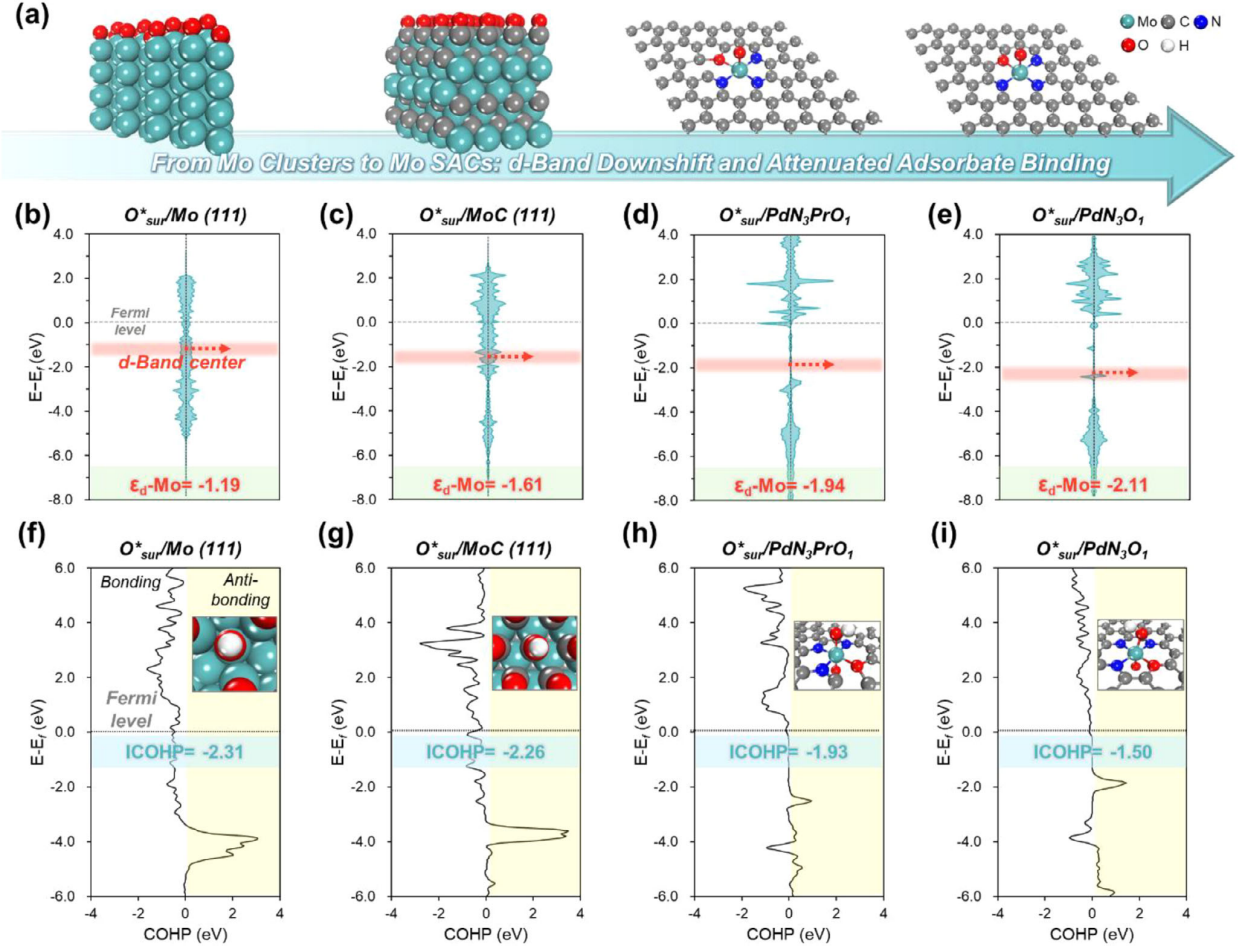
a) Schematic illustration of the structural transition from Mo clusters to SACs, showing the *ε_d_* downshift and moderated adsorbate binding. Partial density of state (PDOS) of b) $O_{sur}^{*}/\mathrm{Mo}$(111), c) $O_{sur}^{*}/\mathrm{MoC}$(111), d) $O_{sur}^{*}/\mathrm{Mo}$@PdN_3_PrO_1_, and e) $O_{sur}^{*}/\mathrm{Mo}$@PdN_3_O_1_, with red arrows indicating the *ε_d_* of Mo active sites. COHP analyses of OH* adsorption on f) $O_{sur}^{*}/\mathrm{Mo}$(111), g) $O_{sur}^{*}/\mathrm{MoC}$(111), h) $O_{sur}^{*}/\mathrm{Mo}$@PdN_3_PrO_1_, and i) $O_{sur}^{*}/\mathrm{Mo}$@PdN_3_O_1_, respectively. ICOHP (integrated crystal orbital Hamilton population) values, where more negative values indicate stronger bonding interactions.

Detailed electronic structure analysis was performed to elucidate the origin of the optimal intermediate binding strength that satisfies the Sabatier principle for Mo SACs. The binding strength of the intermediates on the catalyst surfaces can be explained by the extent of orbital overlap between the active sites and adsorbates, as well as the occupancy of antibonding states within the surface‐adsorbate interaction. These parameters can be quantitatively evaluated using the *ε_d_*,^[^ ^7^, ^8^ ^]^ which reflects the strength of metal–adsorbate orbital overlap, and the crystal orbital Hamilton population (COHP),^[^ ^46^, ^47^ ^]^ which characterizes the bonding and antibonding contributions. Analysis of the *ε_d_* revealed that the pristine SACs (without $O_{sur}^{*}$ passivation: Mo@PdN_3_PrO_1_: −1.03 eV, Mo@PdN_3_O_1_: −1.21 eV) exhibit values comparable to or slightly higher than those of Mo(111) (–1.09 eV) and MoC(111) (–1.43 eV) (Figure S20, Supporting Information). Their proximity to the E*_f_* indicates strong adsorption, in agreement with the high η^ORR^ observed previously (Figure S17, Supporting Information). Following $O_{sur\;}^{*}\mathrm{passivation}$ (**Figure** **5**a‐e), the ε_d_ values of all catalysts shift downward, a phenomenon attributed to surface stabilization that preserves ^*^OOH and optimizes intermediate binding (Figure S18, Supporting Information). Notably, the Mo SACs—$O_{sur}^{*}/\mathrm{Mo}$@PdN_3_PrO_1_ (−1.94 eV) and $O_{sur}^{*}/\mathrm{Mo}$@PdN_3_O_1_ (−2.11 eV)—undergo the most pronounced downshifts, exhibiting significantly lower ε_d_ compared to $O_{sur}^{*}/\mathrm{Mo}$(111) (−1.19 eV) and $O_{sur}^{*}/\mathrm{MoC}$(111) (–1.61 eV). This indicates that systematic electronic restructuring occurred in the $O_{sur}^{*}/\mathrm{Mo}$ SACs, and the resulting substantial downshift moved ε_d_ farther from E*_f_*, promoting optimized intermediate adsorption in accordance with the Sabatier principle and driving Δ*G_dev_* toward zero. It should be noted here that Mo_2_C exhibits a less favorable d‐band center for nitrate reduction compared to MoC, resulting in a higher overpotential requirement (Figure S21, Supporting Information).

A COHP analysis was further conducted to provide a more precise interpretation of adsorption strength, complementing the observed ε_d_ downshift. COHP directly and quantitatively characterizes the OH^*^–active site bonding interaction in the adsorbed intermediate state, which governs the PDS, whereas the ε*_d_* captures the overall adsorption trend from the electronic structure prior to intermediate adsorption. Collectively, the integration of ε_d_ and COHP analyses enables a rigorous and complementary elucidation of adsorption behavior. To this end, energy‐integrated COHP values up to the Fermi level (ICOHP) were calculated for each orbital pair (Figure 5f–i). More negative ICOHP values indicate stronger bonding, whereas values closer to zero indicate weaker interactions. For $O_{sur}^{*}/\mathrm{Mo}$(111) (−2.31 eV) and $O_{sur}^{*}/\mathrm{MoC}$(111) (−2.26 eV), ICOHP values remain strongly negative, reflecting pronounced OH^*^ binding. In contrast, $O_{sur}^{*}/\mathrm{Mo}$@PdN_3_PrO_1_ (−1.93 eV) and $O_{sur}^{*}/\mathrm{Mo}$@PdN_3_O_1_ (–1.50 eV) exhibits less negative values, indicative of more optimal bonding strength in the intermediate state, which directly correlates with the observed reduction in η^ORR^ (Figure S22, Supporting Information). Overall, the combined *ε_d_* and COHP analyses demonstrate that the formation of Mo SACs optimally tunes the electronic structure, allowing $O_{sur}^{*}$ passivation to achieve the ideal *ε_d_*, a state inaccessible in Mo clusters or MoC domains, thereby providing the fundamental origin of the enhanced intrinsic ORR activity observed in Mo_x10 N.

**Figure 6:**
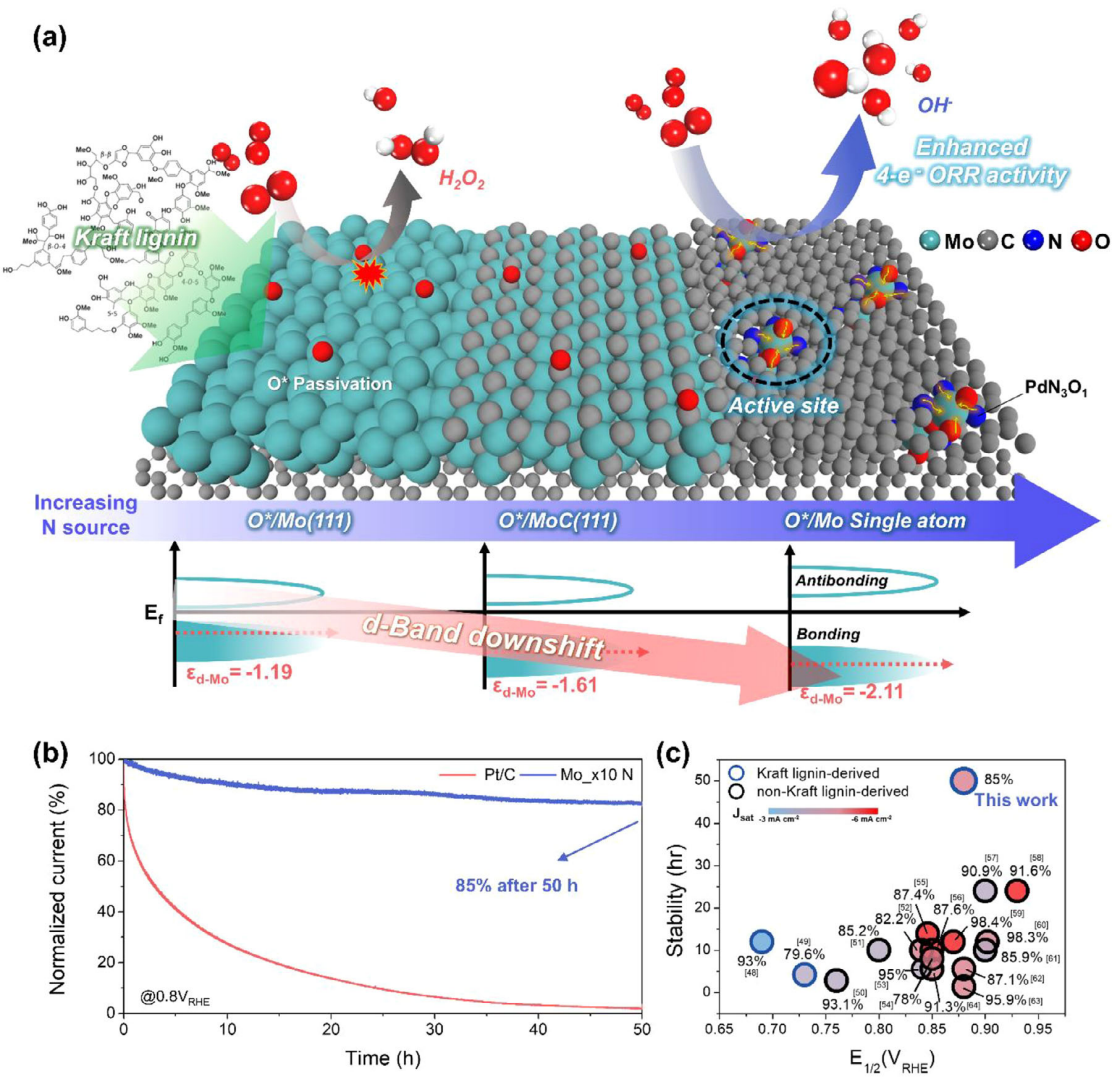
a) Schematic illustration of the transformation from Mo and MoC to atomically dispersed Mo sites within Kraft lignin‐derived carbon, leading to a downshift of the *ε_d_* and enhanced 4e^−^ ORR activity. b) Chronoamperometric stability test at 0.80 V_RHE_ in 1.0 m KOH, showing that Mo_x10 ​N retains ≈85% of its current density after 50 h. c) Benchmark comparison of lignin‐derived ORR catalysts in terms of E_1/2_, J_lim_, and stability.

**Figure** **6**a schematically illustrates that increasing the nitrogen content induces a transition from Mo clusters and MoC nanoparticles to atomically dispersed Mo sites (Mo single‐atom catalyst, Mo SAC), accompanied by a downshift of the *ε_d_* that affects intrinsic ORR activity. Upon conversion to Mo SACs, the surface underwent O^*^ passivation under ORR‐relevant potentials, making $O_{sur}^{*}$/Mo@PdN_3_​O_1_​ the thermodynamically preferred active site. This O^*^‐decorated single‐atom configuration balances intermediate adsorption and raises the OOH^*^ → H_2_​O_2_​ barrier, thereby promoting the enhanced 4e^−^ ORR pathway to H_2_​O compared to the Mo and MoC counterparts. This finding demonstrates that a straightforward, environmentally benign, and scalable synthesis can deliver Mo SAC architectures that intrinsically accelerate the ORR kinetics.

Figure 6b further establishes durability: in 1.0 m KOH at 0.8 V_RHE_​, Mo_x10 ​N retains ≈85% of its initial current density after 50 h, whereas Pt/C undergoes near‐complete deactivation under identical conditions. Given the well‐documented durability limitations of Pt/C—particularly in alkaline media—these data underscore the robustness of Mo_x10 ​N and its potential as a practical Pt alternative for ORR. In addition, such a moderate decrease strongly contradicts the possibility of Mo dissolution–induced activity loss during the SCN^−^ poisoning test in Figure 4g. Instead, the results further support that the observed performance decay arises from the blockage of atomically dispersed Mo sites by SCN^−^ ligands. It should be noted that although a gradual decrease in activity was observed after 50 h of operation, performing detailed ex situ TEM or XPS characterization on the same catalyst layer after the 50‐h stability test is technically challenging, since the RRDE electrode was prepared using a binder‐containing catalyst ink with a low mass loading to ensure reliable electrochemical measurements. The strong adhesion of the binder and the small catalyst quantity make it practically impossible to retrieve the film without introducing mechanical or chemical artifacts that could distort the intrinsic structure. In future work, in situ or operando spectroscopic techniques (e.g., XAS, Raman, or XPS under electrochemical conditions), as well as device‐level investigations in integrated fuel‐cell or metal–air battery configurations, will be valuable for further elucidating the origin of performance decay and exploring strategies to enhance the long‐term durability of Mo‐based single‐atom catalysts. In comparison with previously reported lignin‐derived ORR catalysts, the E_1/2_​ and J_lim_​ of Mo_x10 ​N do not exceed the very best values obtained from non‐Kraft, more highly refined lignin precursors (Figure 6c).^[^ ^48^, ^49^, ^50^, ^51^, ^52^, ^53^, ^54^, ^55^, ^56^, ^57^, ^58^, ^59^, ^60^, ^61^, ^62^, ^63^, ^64^ ^]^ Nevertheless, Kraft lignin is the least processed; thus, it is inexpensive and widely available. In this context, Mo_x10 ​N delivers activity that ranks among the leading lignin‐based results, validating the practical merit of the approach. Leveraging Kraft lignin as the carbon precursor confers clear economic and environmental advantages; relative to widely used carbon supports such as carbon black (typically retailing at US$55–100 per 50 g), bulk Kraft lignin is available at US$0.25–0.50 kg^−1^, corresponding to a raw‐material cost reduction well exceeding 90%.^[^ ^65^ ^]^ A broader benchmark against Mo‐based catalysts in 0.1 m KOH is summarized in Table S2 (Supporting Information). Although Mo_x10 ​N does not set the absolute record in E_1/2_​ or J_lim_​, its combination of low‐cost feedstock and simple synthesis positions it as a compelling ORR catalyst. Consequently, the valorization of Kraft lignin as a carbon support not only reduces cost and advances sustainability but, together with the benchmark durability of Mo_x10 ​N relative to other lignin‐derived systems, marks a key milestone toward the practical application of biomass‐derived ORR electrocatalysts.

## 3 Conclusion

In summary, we demonstrated a sustainable and cost‐effective strategy for fabricating Mo single‐atom catalysts by the valorization of Kraft lignin as a low‐cost and abundant carbon matrix. This synthesis enabled the controlled transformation of Mo clusters and Mo carbide domains into atomically dispersed Mo sites, coupled with an optimized electronic structure. Experimental and theoretical analyses revealed that the enhanced intrinsic ORR activity of Mo_x10 N originates from balanced intermediate adsorption at the Mo SACs, driven by the synergistic effect of single‐atom dispersion and electronic modulation. The catalyst achieved a half‐wave potential of 0.88 V_RHE_, excellent four‐electron selectivity, and benchmark durability, positioning it among the most competitive lignin‐derived ORR electrocatalysts. Importantly, the use of Kraft lignin not only delivers high catalytic performance but also advances the valorization of an underutilized industrial byproduct, underscoring both economic and environmental benefits. This study establishes a milestone in the design of biomass‐derived SACs and provides a promising platform for practical applications in sustainable energy conversion systems.

## 4 Experimental Section

### Materials

Kraft lignin (provided by West Fraser Corp., Hinton Mill, Alberta, Canada), Sodium molybdate dihydrate (Na_2_MoO_4_·2H_2_O, ACS reagent, ≥99%, Sigma–Aldrich), zinc nitrate hexahydrate (Zn(NO_3_)2·6H_2_O, reagent grade, 98%, Sigma–Aldrich), dicyandiamide (DCD, 99%, Sigma–Aldrich), sodium hydroxide (NaOH, powder, reagent grade, 97%, Sigma–Aldrich), and potassium hydroxide (KOH, flakes, reagent grade, 90%, Sigma–Aldrich) sodium thiocyanate (NaSCN, ≥99.99% trace metals basis, Sigma–Aldrich), and 1xPBS, sterile‐filtered, Thermo Scientific, Alfa Aesar) were used as received without further purification.

### Fabrication of the Electrocatalysts

Kraft lignin (0.32 g) was dissolved in 40 mL of deionized (DI) water containing NaOH (0.05 g) under continuous stirring for 15 min. Sodium molybdate dihydrate and zinc nitrate hexahydrate were then added at a molar ratio of 1:5 (Mo:Zn), followed by stirring at 60 °C for 1 h. The resulting Mo/Zn–lignin (Mo/Zn–L) complex was aged for 12 h, collected by centrifugation, and dried overnight at 80 °C. The dried Mo/Zn–L was thoroughly ground with DCD (10× by weight) to form a homogeneous mixture, which was then carbonized in a tube furnace under Ar flow. The heating profile was 550 °C for 1 h followed by 1000 °C for 1 h, at a ramp rate of 5 °C min^−1^. The product was washed twice with DI water and ethanol, and dried overnight at 60 °C.

### Material Characterizations

Surface functional groups of lignin were characterized by Fourier‐transform infrared spectroscopy (FT‐IR, Nicolet iS10, Thermo Scientific, ATR mode). Elemental analysis (EA; FLASH 2000 CHNS, Thermo Scientific) determined elemental composition. Morphology and particle distribution were examined using field‐emission scanning electron microscopy (FE‐SEM, Inspect F, FEI) and transmission electron microscopy (TEM, Titan 300 kV, FEI), including HAADF‐STEM and EDS mapping. Crystalline structures were analyzed by X‐ray diffraction (XRD; D/MAX 2500, Rigaku) using Cu Kα radiation (λ = 1.5406 Å). Surface chemical states were probed by X‐ray photoelectron spectroscopy (XPS; Nexsa, Thermo Fisher Scientific) with a monochromated Al Kα source (1486.6 eV). Oxidation states and local coordination environments were investigated by X‐ray absorption spectroscopy (XAS) at the 10C beamline, Pohang Accelerator Laboratory (PAL), Republic of Korea.

### Electrochemical Measurements

Electrocatalytic performance was measured on a BioLogic VSP workstation using a standard three‐electrode setup. A catalyst‐coated glassy carbon rotating disk electrode (RRDE, geometric area 0.2468 cm^2^, Pine Instruments) served as the working electrode. An Hg/HgO electrode (1.0 m NaOH) and a graphite rod were used as reference and counter electrodes, respectively. All potentials were converted to the reversible hydrogen electrode (RHE) scale using:

(1) $$E_{\mathrm{RHE}}=\mathrm{ESCE}\;+0.0591\;\times \mathrm{pH}\;+\;0.140V$$

### Ink Preparation

For Mo catalysts, 11 mg of catalyst (2.14 wt.% Mo, determined by ICP) was dispersed in 2.49 mL isopropanol, 2.49 mL DI water, and 20 µL of 5 wt.% Nafion solution, then ultrasonicated for 20 min. For Pt/C (20 wt.%), 1.18 mg was dispersed in the same solvent composition to match the metal content of the Mo ink. Catalyst inks (80 µL) were drop‐cast onto the polished RRDE, yielding loadings of 0.73 mg cm^−^^2^ (Mo) and 0.0765 mg cm^−^^2^ (Pt/C), corresponding to ≈15.3 µg cm^−^^2^ metal in both cases.

### Measurements

The electrolyte (1.0 m KOH) was purged with N_2_ or O_2_ for ≥20 min, with continuous O_2_ bubbling during ORR and CA tests. Linear sweep voltammetry (LSV) was performed from 1.10 to −0.30 V_RHE at 10 mV s^−1^. Background LSVs (N_2_‐saturated) were subtracted to obtain corrected ORR curves. Cyclic voltammetry (CV) was recorded between 0.40 and 1.00 V_RHE at 50 mV s^−1^ until stable curves were achieved. Stability was assessed by chronoamperometry (CA) at 0.8 V_RHE for 50 h in O_2_‐saturated 1.0 m KOH at 1600 rpm.

### SCNH Poisoning Tests

Conducted in 1× PBS containing 5 mm NaSCN. ORR polarization curves before and after NaSCN addition were compared to evaluate active‐site blocking. Capacitive currents were subtracted, and all current densities were normalized to geometric electrode area. All measurements were repeated at least three times for reproducibility.

### RRDE Analysis

The H_2_O_2_ yield and electron transfer number (n) were calculated as:

(2) $$\mathrm{OO}H^{-}\%=200\frac{I_{r}/N}{\left|I_{d}\right|+I_{r}/N}$$

(3) $$n=4\frac{I_{d}}{I_{d}+I_{r}/N}$$

where I_d_ and I_r_ denote the disk and ring currents, respectively, and N was the collection efficiency of the Pt ring (0.37).

### Computational Details

All ab initio calculations were performed using the Vienna Ab initio Simulation Package (VASP 5.4.4).^[^ ^66^, ^67^, ^68^, ^69^ ^]^ The projector augmented wave (PAW) method^[^ ^70^, ^71^ ^]^ was used with a generalized gradient approximation based on the Perdew–Burke–Ernzerhof (PBE) ^[^ ^72^ ^]^ functional. A plane‐wave cut‐off energy of 500 eV was used. The lattice constants and internal atomic positions were fully optimized until the residual forces were less than 0.04 eV  Å^−1^. Integration over the Brillouin zone was performed using the Monkhorst‐Pack scheme with a 3 × 3 × 1 k‐point mesh for the Mo, MoC, Mo@PdN_3_O_1,_ and Mo@PdN_3_PrO_1_ structures. To account for non‐bonding interactions, the DFT‐D3 ^[^ ^73^ ^]^ dispersion correction was applied to all structures. To construct the surface models of Mo and MoC, their crystal lattices were cleaved along the representative low‐index (111) facet based on the HRTEM observations in Figure 2b. A vacuum spacing of 20 Å was applied along the *z*‐direction to avoid spurious interactions between periodic images. During relaxation, the bottom two layers were fixed to mimic the bulk characteristics, whereas the remaining atoms were fully relaxed. Thus, these slab models were used to represent the Mo(111) and MoC(111) surfaces. COHP analysis was used to understand the chemical bonds by dividing the electronic structure into bonding and antibonding contributions using the local orbital basis suite toward electronic structure reconstruction (LOBSTER) code based on the pbeVaspFit2015 basis set. Moreover, as a descriptor for numerically evaluating the chemical bond strength, the integrated COHP value (ICOHP) up to the E*_f_* was considered.

## Conflict of Interest

The authors declare no conflict of interest.

## Author Contributions

J.P., J.P., and J.H.S. contributed equally to this work. J.P. conducted the experiments, J.P. analyzed the data, and wrote the manuscript. J.H.S. conducted DFT computational calculations and helped with manuscript preparation. J.S.B.C contributed to the DFT computational calculations. H.‐S.O., E.‐D.K., Y.‐J.K., and Y.K. contributed to the experiments. G.S. and M.J.K. contributed to the data analysis. H.‐S.B., H.S.P., and C.‐J.Y. helped with manuscript preparation. S.U.L. contributed to the DFT calculation and contributed to the data analysis. H.‐S.O., K.H.K., and W.Y. supervised the project, directed the research, and contributed to the manuscript.

## Supporting information



Supporting Information

## Data Availability

The data that support the findings of this study are available from the corresponding author upon reasonable request.
