# Supplementary material for: Modulation of Electronic Structure in Kraft Lignin‐Derived Mo Single‐Atom Catalysts for Optimized Electrochemical Oxygen Reduction
Source: Adv Sci (Weinh). 2025 Dec 12;13(12):e22273. doi: 10.1002/advs.202522273 (PMC12948250; doi:10.1002/advs.202522273)
Supplement: Supplementary file 1 — Supporting Information [file ADVS-13-e22273-s001.docx]

Modulation of Electronic Structure in Kraft Lignin-Derived Mo Single-Atom Catalysts for Optimized Electrochemical Oxygen Reduction

Junbeom Park^‡^, Jaemin Park^‡^, Jun Ho Seok^‡^, Ji Soo Byun, Cheoulwoo Oh, Eung-Dab Kim, Young-Jin Ko, Youngeun Kim, Gawon Sim, Min Jae Kim, Hyeon-Seok Bang, Ho Seok Park, Chun-Jae Yoo, Sang Uck Lee, Hyung-Suk Oh^*^, Kwang Ho Kim^*^, Wooseok Yang^*^

*
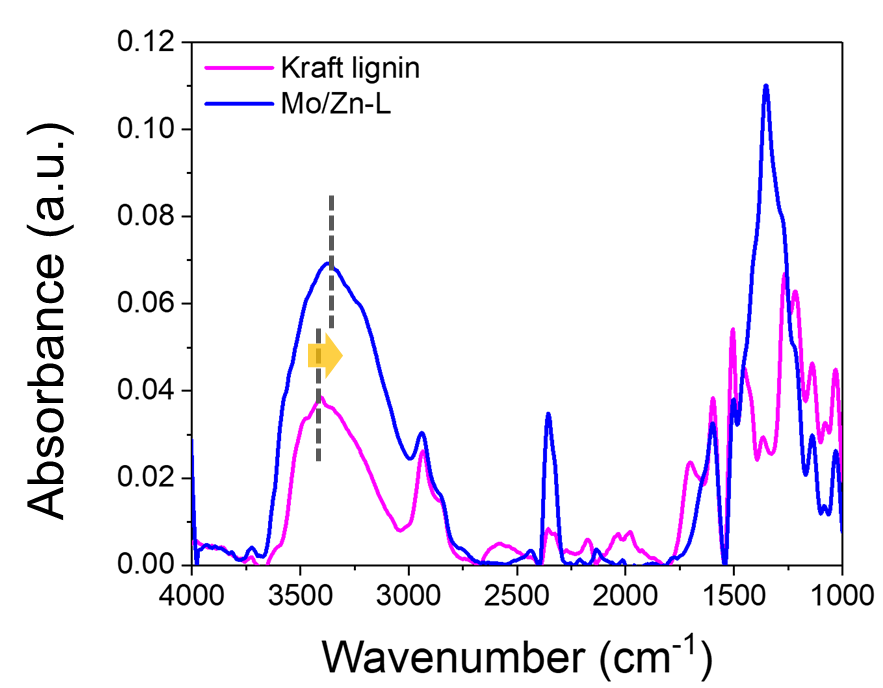
*

**Figure S1.** Attenuated total reflection Fourier-transform (ATR-FTIR) infrared spectra of pristine Kraft lignin and as-prepared metal–lignin precursor (Mo/Zn-L).

^
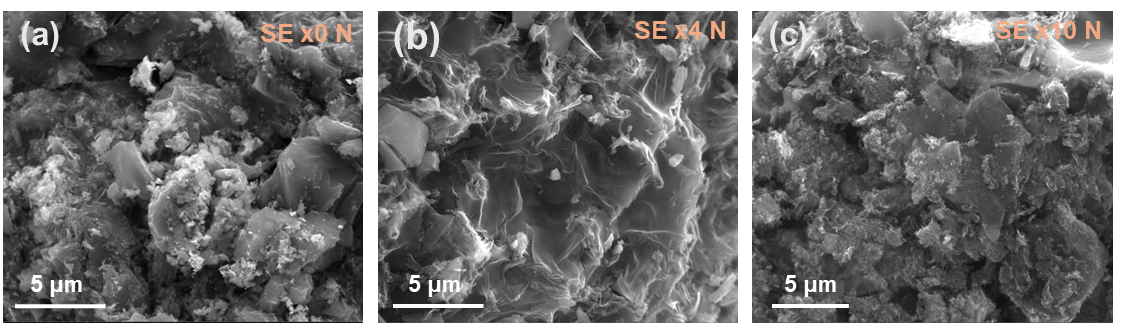
^

**Figure S2.** SEM images of (a) Mo x0 N, (b) Mo x4 N, and (c) Mo x10 N.


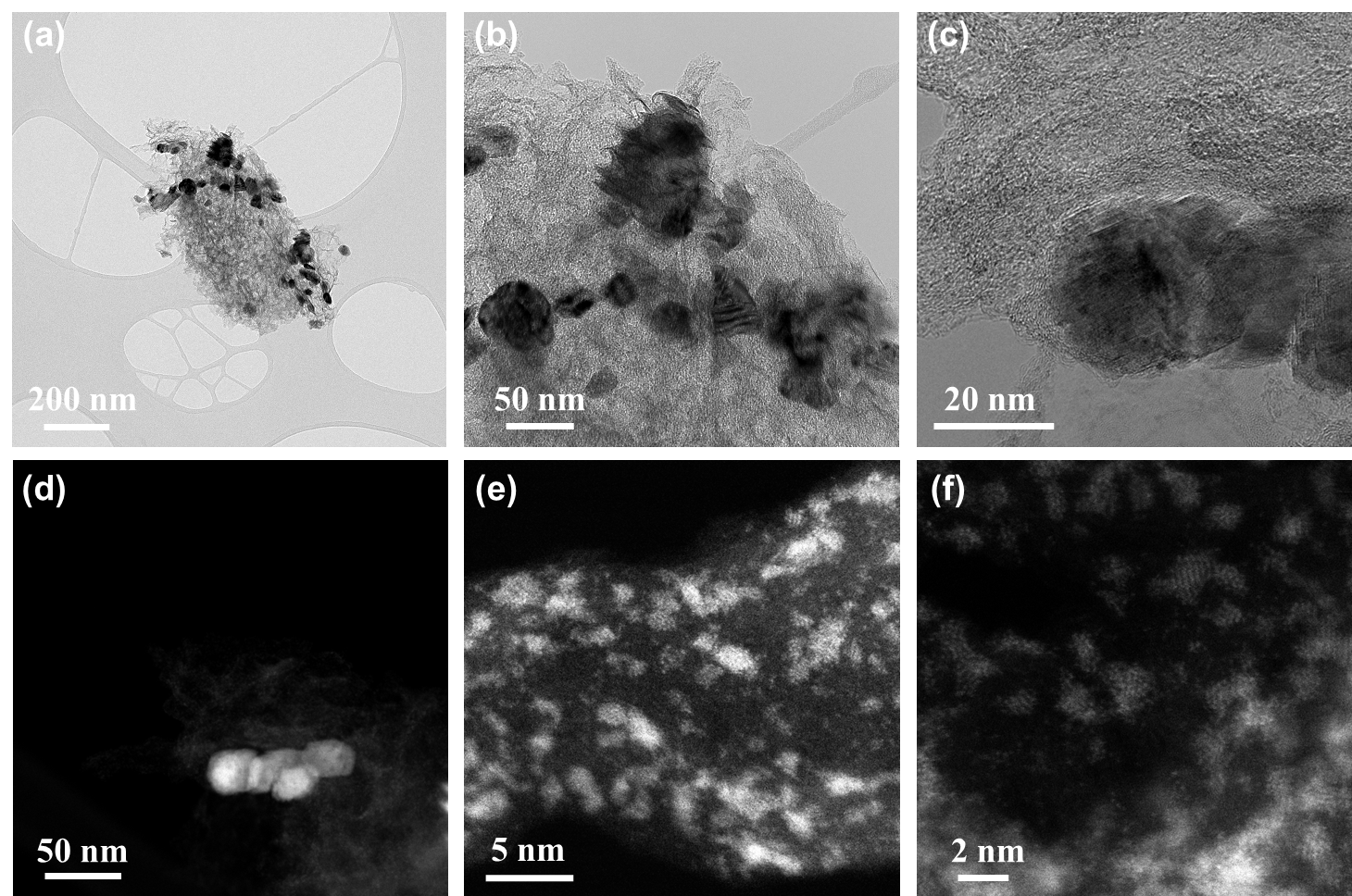


**Figure S3.** Low-magnification TEM image and HAADF-STEM images of Mo x0 N.


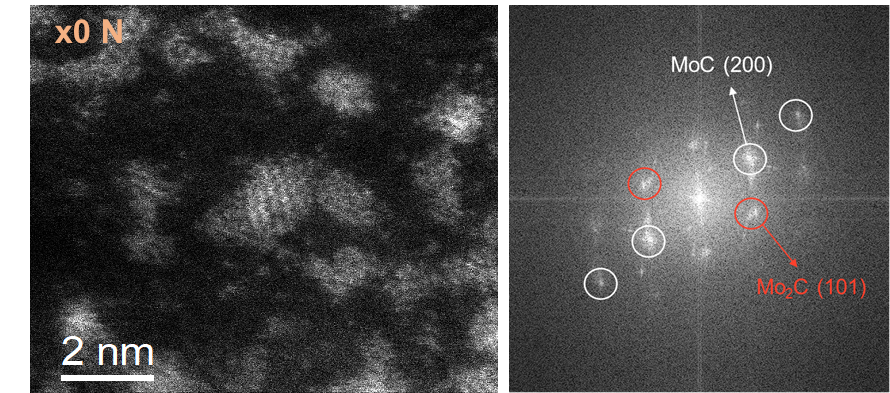


**Figure S4**. High-resolution TEM (left) and corresponding fast Fourier transform (FFT) pattern (right) of the Mo_2_C.


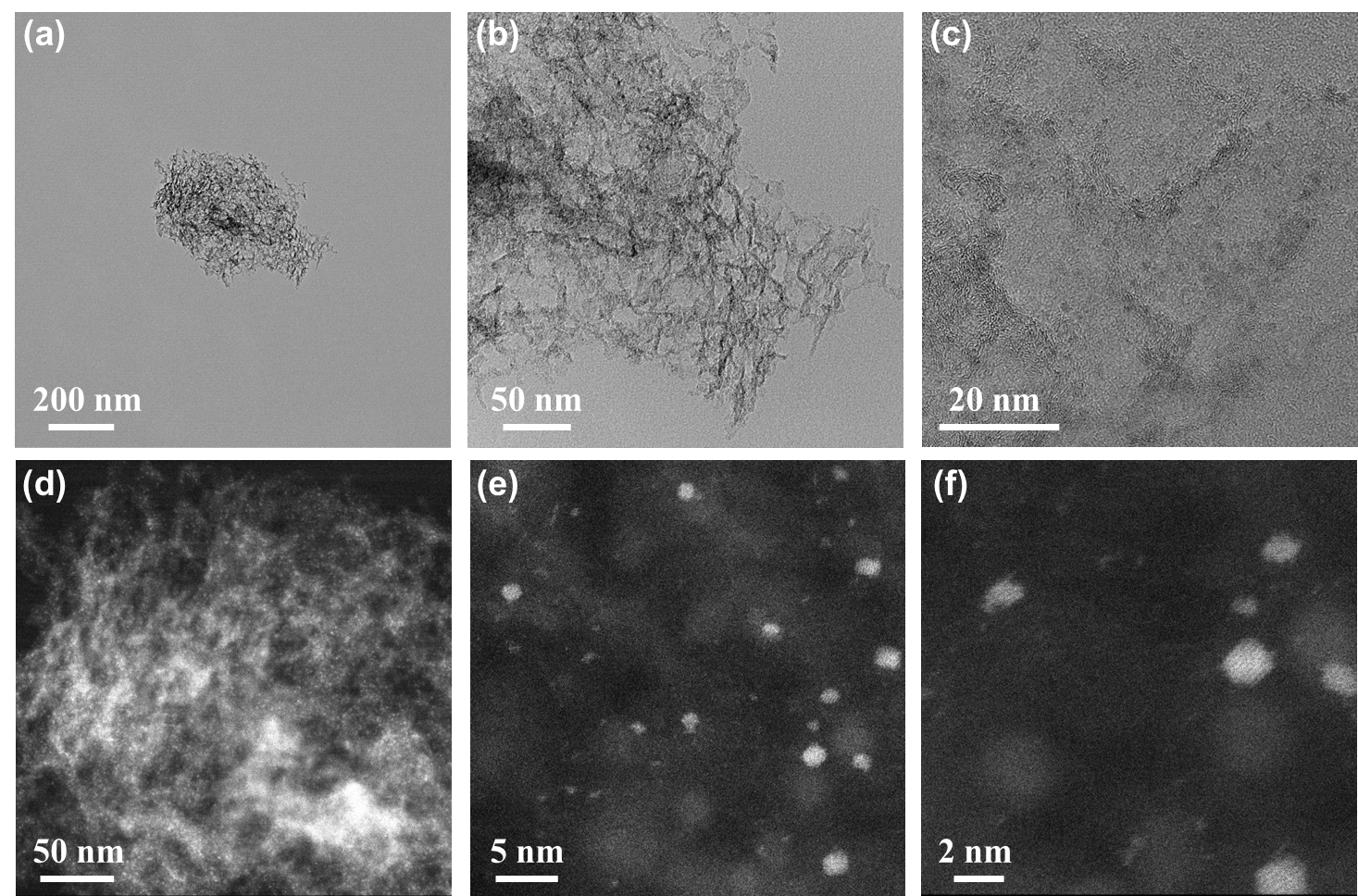


**Figure S5.** Low-magnification TEM image and HAADF-STEM images of Mo x4 N.


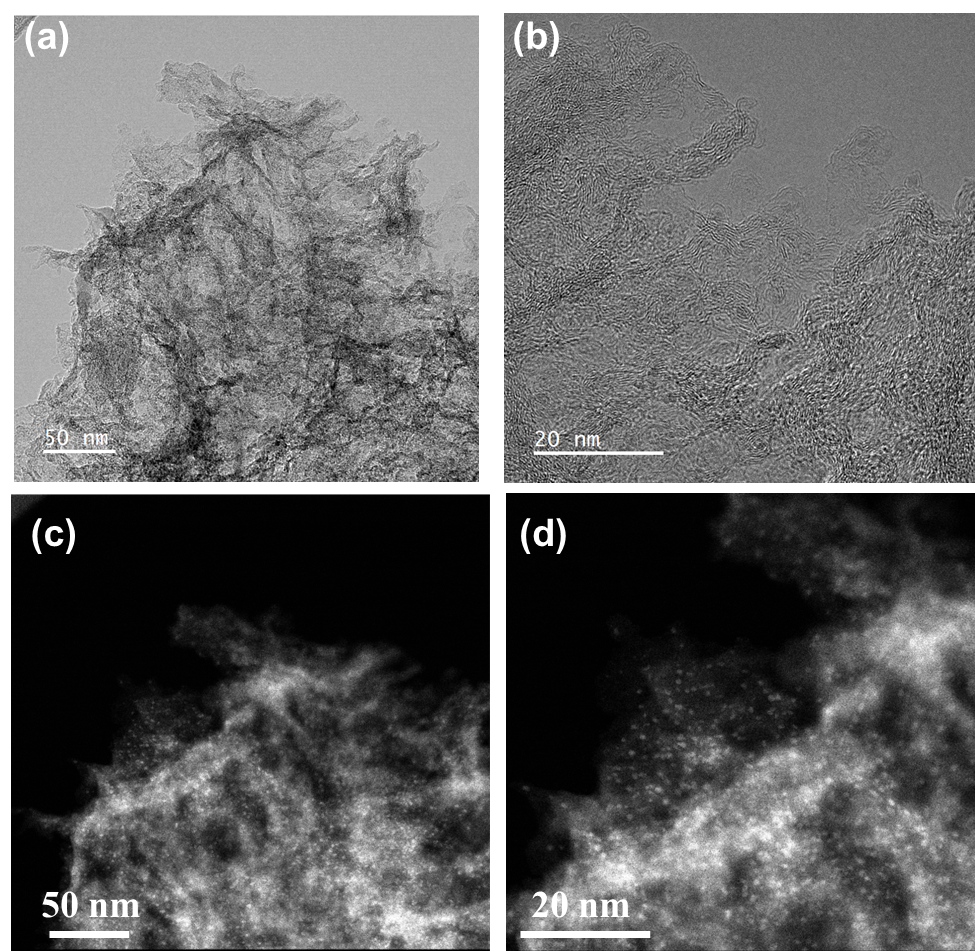


**Figure S6.** Low-magnification TEM image and HAADF-STEM images of Mo x10 N.

**
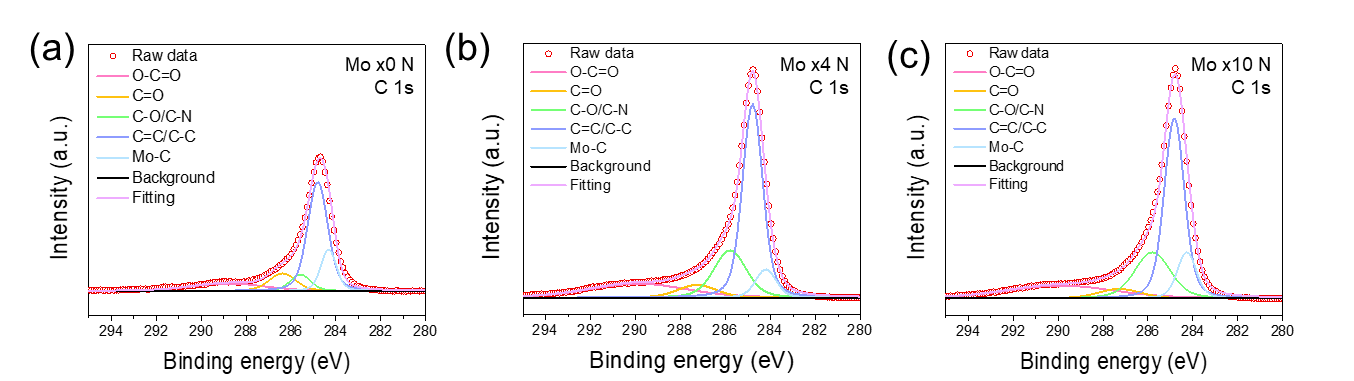
**

**Figure S7.** High-resolution XPS spectra and peak deconvolution for the C 1s regions of Mo x0 N, Mo x4 N, and Mo x10 N.

**
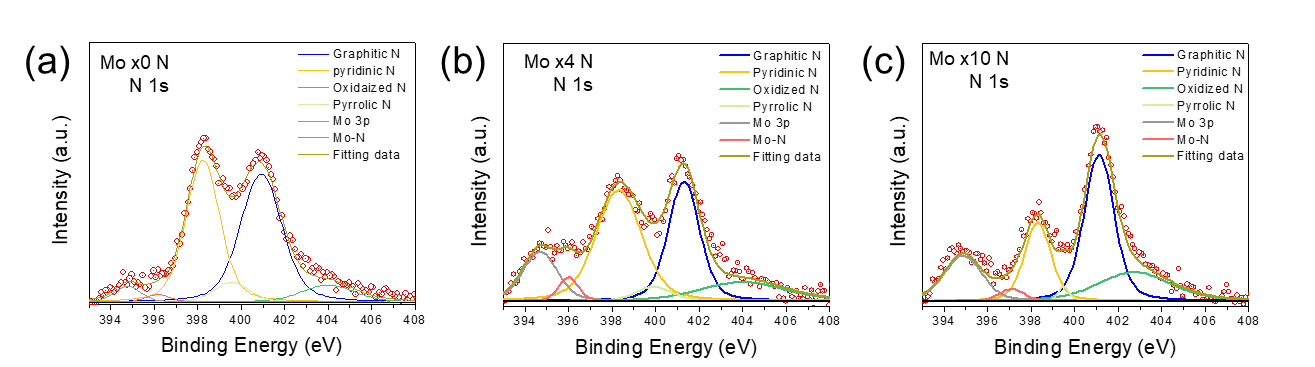
**

**Figure S8.** High-resolution XPS spectra and peak deconvolution for the N 1s regions of Mo x0 N, Mo x4 N, and Mo x10 N.


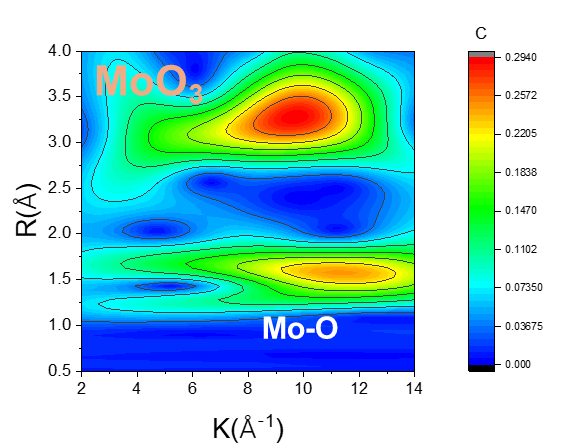


**Figure S9.** The Wavelet-transform (WT) contour plots of EXAFS signals for MoO_3._


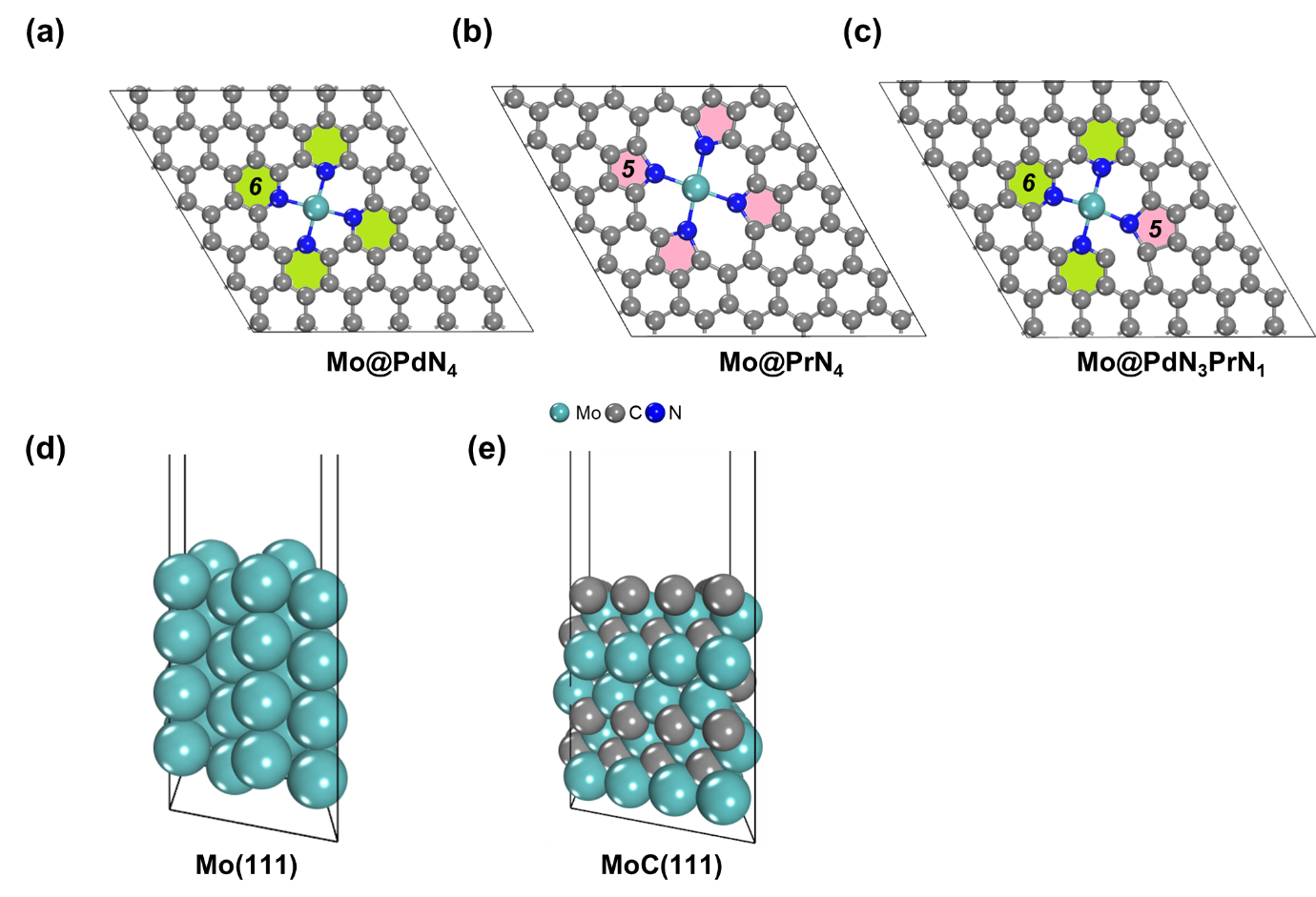


**Figure S10**. (a) Mo@PdN_4_ with a Mo SAC coordinated by four pyridinic N, (b) Mo@PrN_4_ with four pyrrolic N, (c) Mo@PdN_3_Pr_1_ with three pyridinic and one pyrrolic N, representing Mo_x10N. (d) Mo(111) and (e) MoC(111) surface structures, representing Mo_x0N.


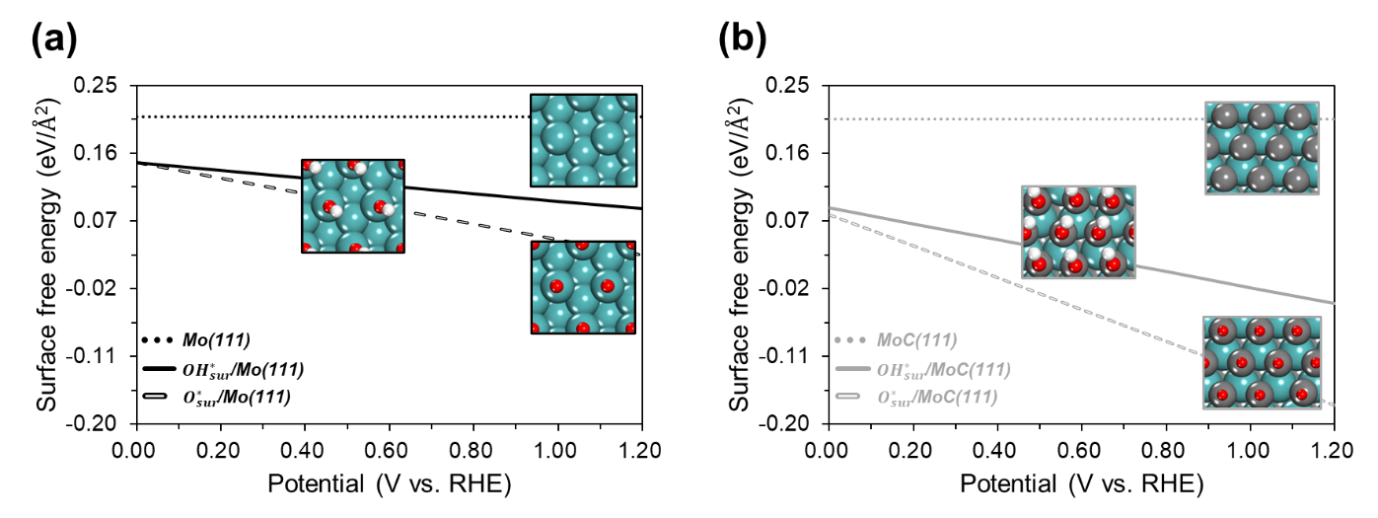


**Figure S11**. (a) Calculated surface free energies as a function of the applied potential (U vs. RHE) for (a) Mo(111) and (b) MoC(111) in clean, $O_{sur}^{*}$, and ${OH}_{sur}^{*}$.

**
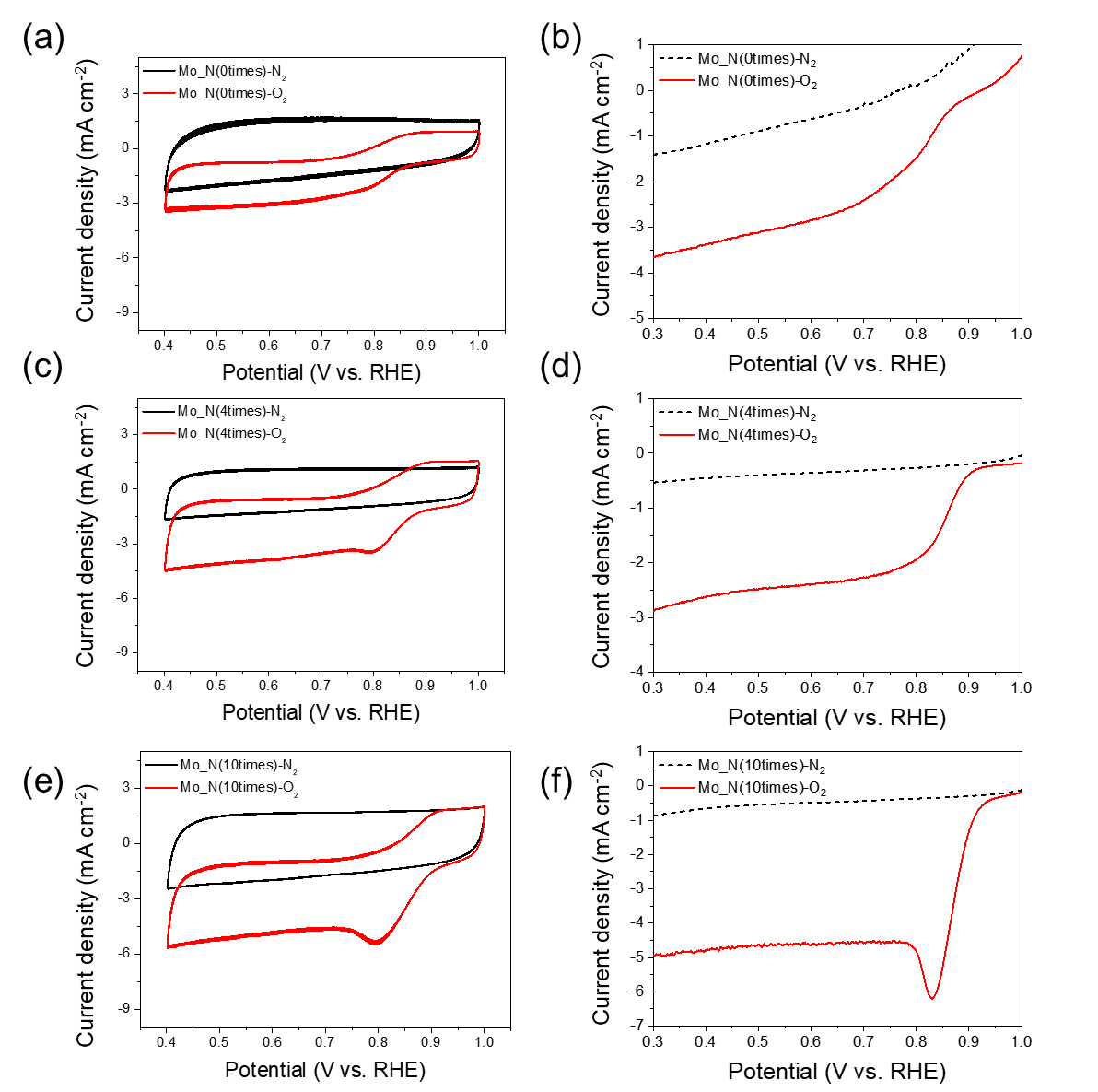
**

**Figure S12.** Cyclic-voltammetry profiles (left column) and linear-sweep polarization curves (right column) recorded in N₂ and O_2_-saturated electrolyte, respectively for (a, b) Mo x0 N, (c, d) Mo x4 N, and (e, f) Mo x10 N.

**Figure S13.** The summary of half-wave potentials (E_1/2_​) and diffusion-limited current densities (J_lim_​) for Pt/C, Mo_x0 N, and Mo_x10 N.

**Figure S14.** ORR polarization curves for multiple Mo x10 N samples for reproducibility.

**
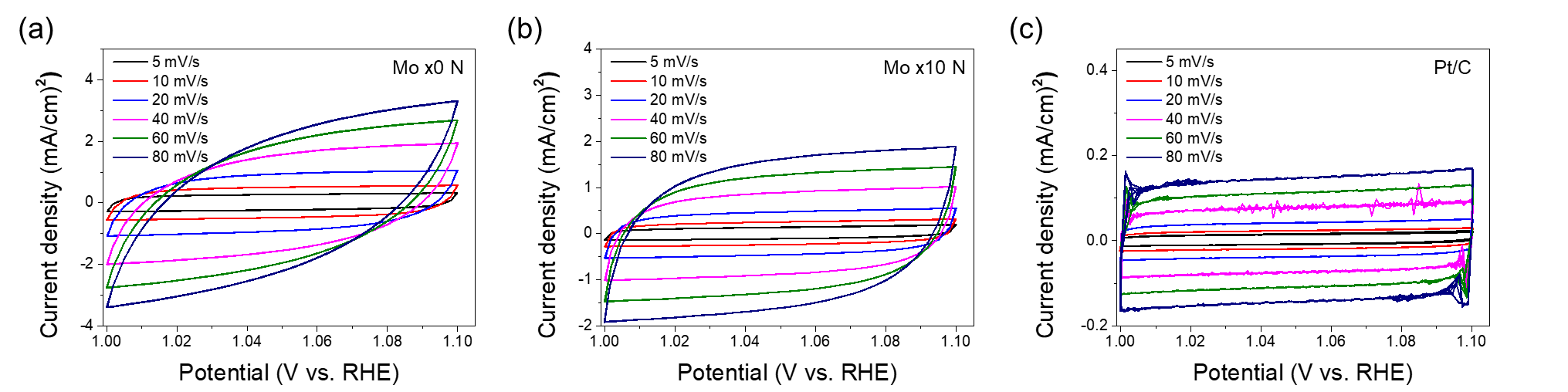
**

**Figure S15.** Cyclic-voltammetry profiles of (a) Mo x0 N, (b) Mo x10 N, and (c) Pt/C, (recorded in N₂-saturated 1 M KOH at scan rates 5,10, 20, 40, 60, and 80 mV s⁻¹ (color-coded, see legends).

**Figure S16.** The specific activity (J_s_​, current density normalized to ECSA) of Mo_x0 ​N and Mo_x10 ​N.


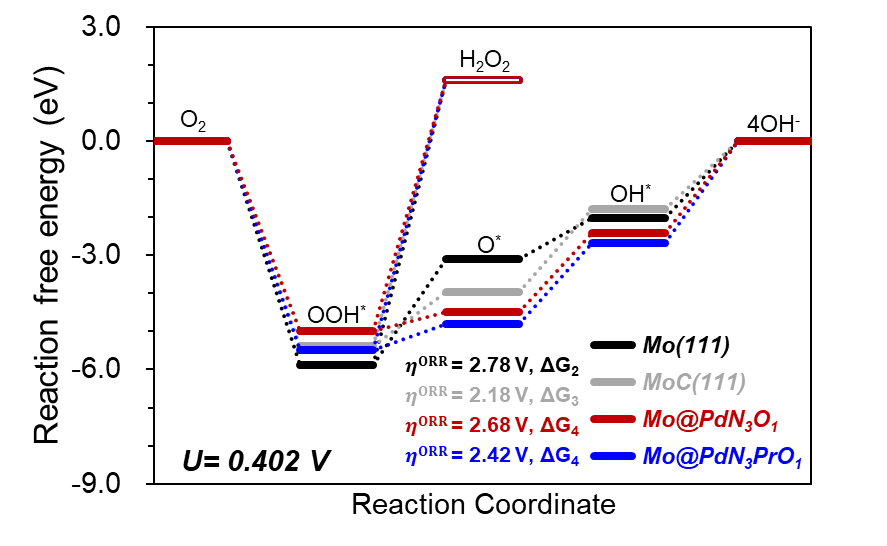


**Figure S17.** Free-energy diagram (FED) for (ORR) on Mo(111), MoC(111), Mo@PdN_3_O_1_, and Mo@PdN_3_PrO_1_ at U = 0.402 V in alkaline media.


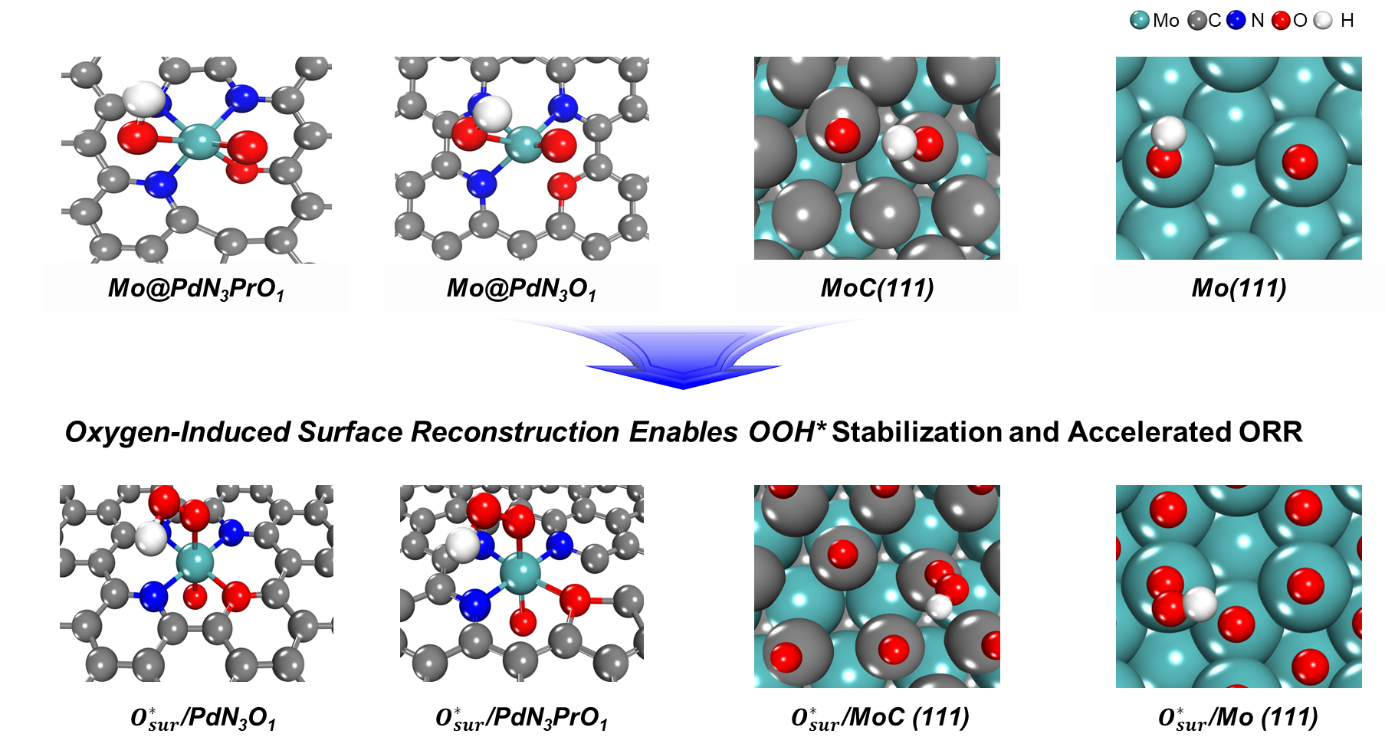


**Figure S18.** Schematic illustration comparing OOH* adsorption on bare versus $O_{sur}^{*}/$Mo-based catalysts (Mo@PdN₃PrO₁, Mo@PdN₃O₁, MoC(111), Mo(111)). On clean surfaces, OOH* readily dissociates into OH*/O*, leading to unstable adsorption. In contrast, $O_{sur}^{*}$ stabilizes the catalysts, maintaining adsorbed OOH* and thereby facilitating the efficient ORR pathway.


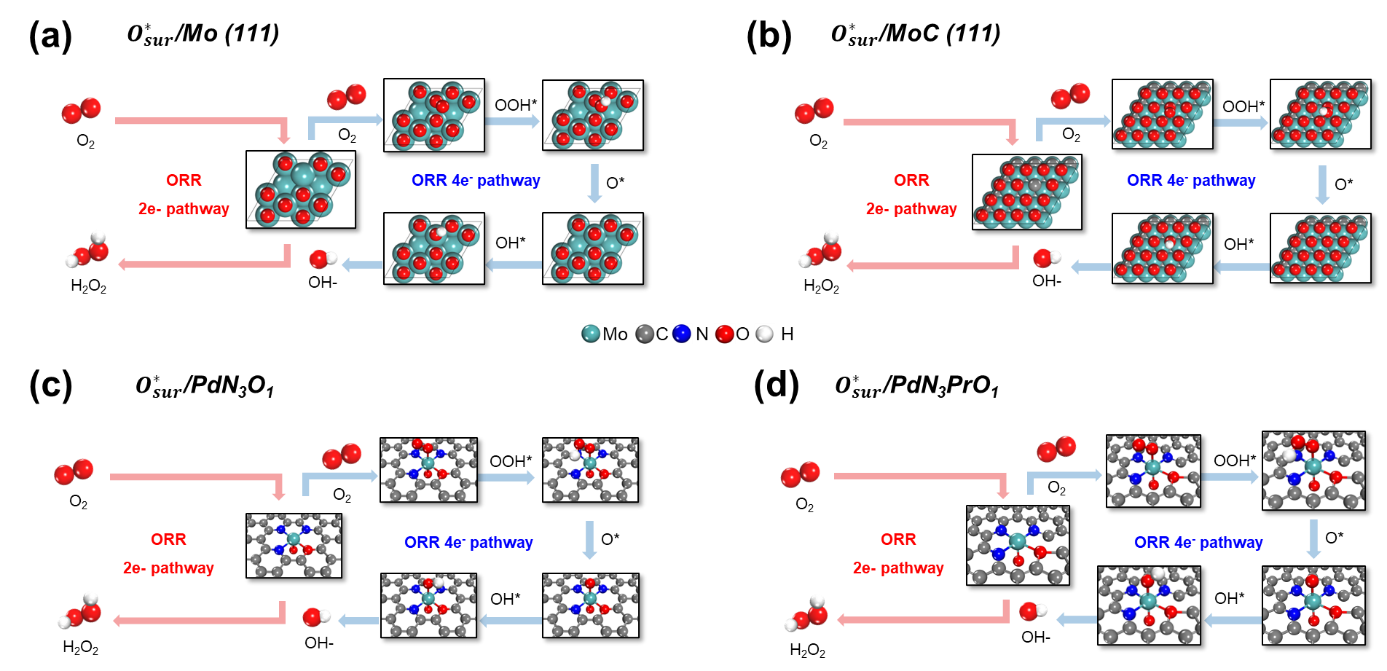


**Figure S19**. Schematic reaction-coordinate diagrams for ORR on oxygen-covered catalysts. (a) $O_{sur}^{*}$/Mo(111), (b) $O_{sur}^{*}$/MoC(111), (c) $O_{sur}^{*}$/Mo@PdN_3_O_1_, (d) $O_{sur}^{*}$/Mo@PdN_3_Pr_1_. The left (pink) branch depicts the ORR 2e^-^ pathway, whereas the right (blue) branch depicts the four-electron pathway.


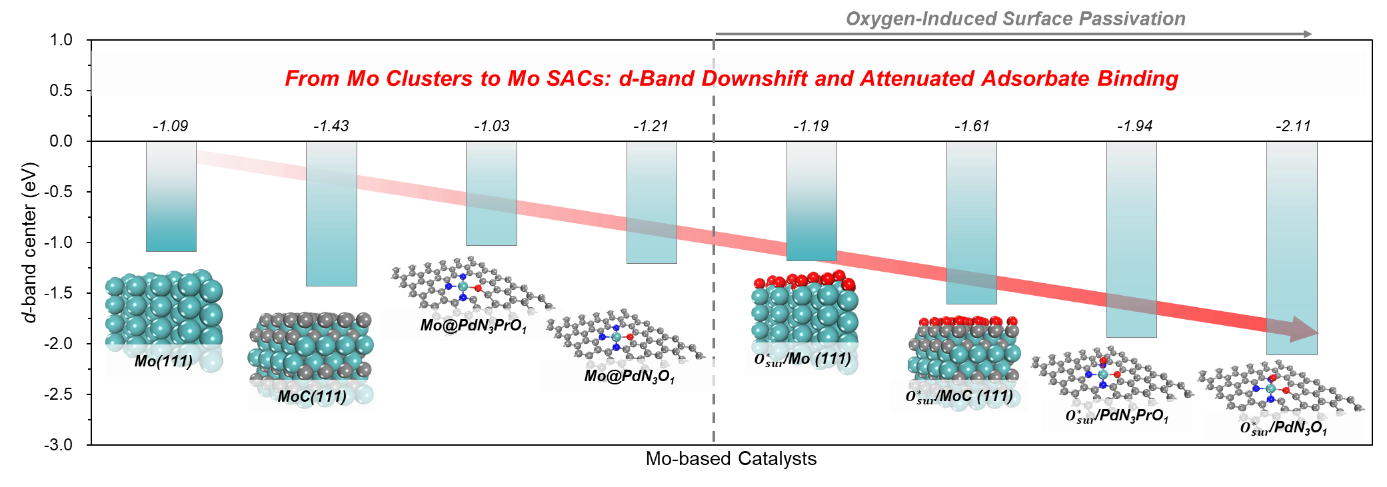


**Figure S20.** Variation of the Mo *d*-band center (*ε_d_*) for Mo(111), MoC(111), Mo@PdN_3_PrO_1_, and Mo@PdN_3_O_1_, together with their $O_{sur}^{*}$counterparts. A progressive downshift of *ε_d_* is observed toward $O_{sur}^{*}$/Mo SACs, reflecting oxygen passivation and attenuated adsorbate binding.


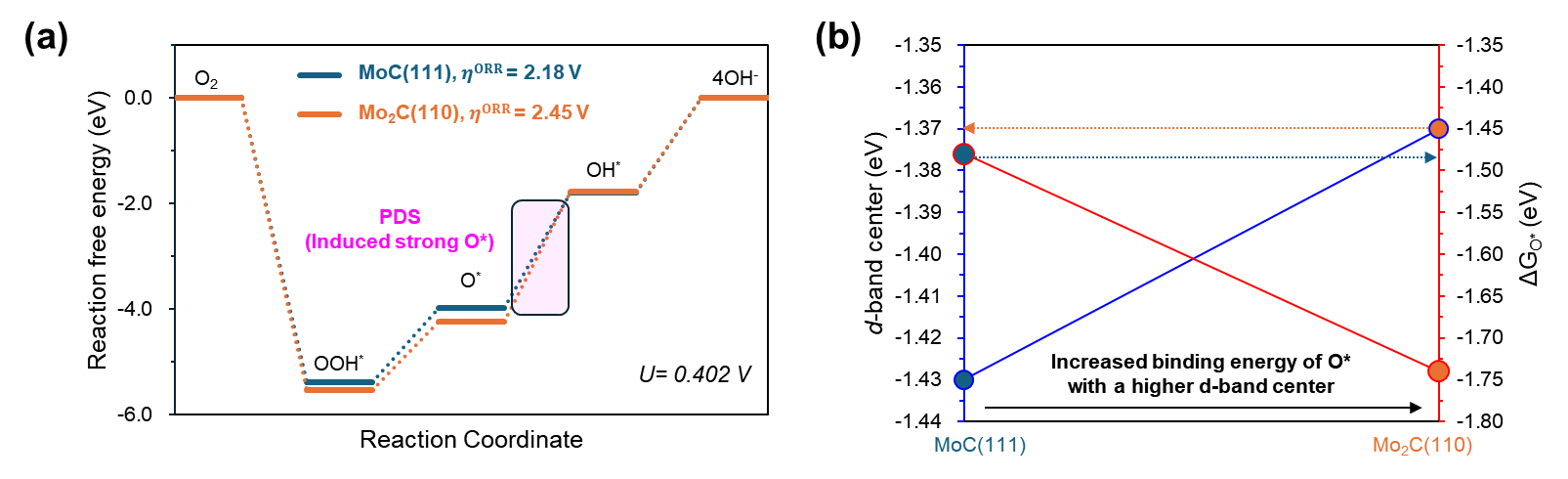


**Figure S21**. (a) FED for ORR on MoC(111), Mo_2_C(110) (b) Binding free energies of oxygen (ΔG_O*_ ) and *d*-band center values of the MoC(111), and Mo_2_C(110)


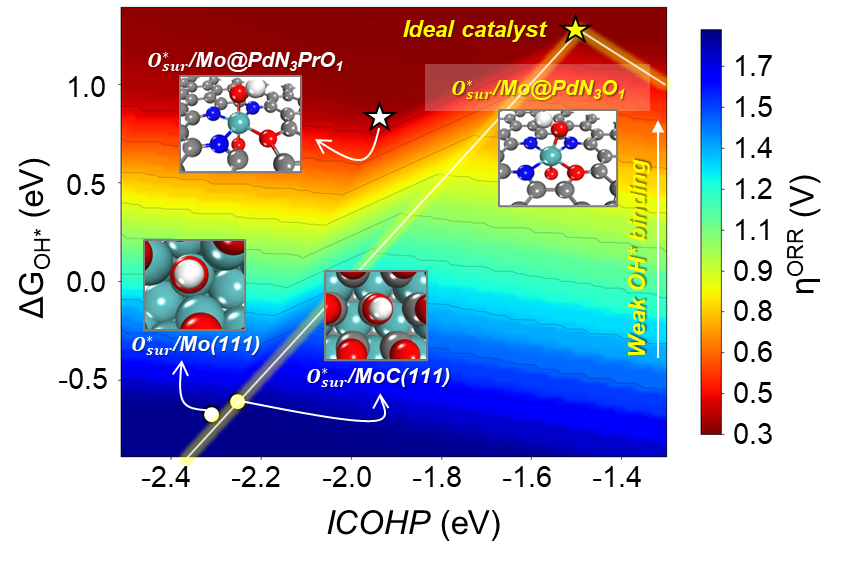


**Figure S22**. Contour plot shows the relationship between the Gibbs free energies of OH (${\Delta G}_{OH*}$) and ICOHP values of the $O_{sur}^{*}/$Mo-based catalysts. The transition from Mo clusters to atomically dispersed Mo SACs optimizes adsorption strength, thereby significantly boosting ORR activity.

**Note S1. Formation energy of Mo SAC catalysts.**

To assess the thermodynamic viability of the modified Mo SAC catalysts, we computed the formation energy per atom as

|  | $E_{for}= \frac{E_{catalyst}-(n_{c}\mu_{c}+n_{N}\mu_{N}+n_{O}\mu_{O}+n_{Mo}\mu_{Mo})}{A}$ | Eqn. S1 |
| --- | --- | --- |

, where $E_{catalyst}$ is the DFT total energy of the relaxed Mo/N/O-modified gaphene supercell; $n_{i}$ denotes the number of atoms of species *i* (*i* = C, N, O, Mo); and $A$ is the total atom count in the unit cell. The chemical potentials $\mu_{i}$ are taken as follows: $\mu_{c}$ is the energy per carbon atom in pristine graphene, $\mu_{n}= \frac{1}{2}E\left( N_{2} \right)$, $\mu_{O}= \frac{1}{2}E\left( O_{2} \right)$ and $\mu_{Mo}$ is the energy per atom of bulk molybdenum in its stable bcc phase.

**Note S2. Comparison of catalyst stability in ORR operation conditions by DFT calculation.**

To compare the stability of the oxygen species (O and OH^-^) of Mo-based catalysts in ORR operation conditions, we evaluated the sustainability of Mo(111), MoC(111), Mo@PdN_3_O_1_, and Mo@PdN_3_PrO_1_ catalysts based on calculating surface-free energy ($\sigma(U)$) as a function of the applied potential (U).

|  | $\sigma\left( U \right)= \sigma_{for}+\frac{G_{catalyst}^{O and OH\left( n \right)}-(G_{catalyst}+(n)*E_{O and OH})-neU}{A}$ | Eqn. S2 |
| --- | --- | --- |

, where $\sigma_{for}$ is the formation energy of catalysts, *n* is the number of adsorbed oxygen species (OH*, O*) in the system, $G_{catalyst}^{O and OH\left( n \right)}$ is the adsorption free energy of *n* oxygen species adsorbed catalysts, $G_{catalyst}$ is the Gibbs free energy of the catalyst, *e* is the electron charge, and *A* is the surface area.

**Note S3. Evaluating ORR activity in alkaline media using the DFT perspective**

In this work, we used the theoretically well-defined free energy diagram (FED) approach proposed by Norskov group. It has been generally accepted for use in electrochemical studies based on density functional theory (DFT) calculations. To investigate catalytic reactions on specific surface structures, we generally employ the thermodynamic stability of the intermediates as the main descriptor, which determines the catalytic performance. In the case of oxygen reduction reaction (ORR), the four-electron reaction has been generally accepted, where this process in an alkaline environment can be described as follows:

| O_2_* + H_2_O(l) + e^-^ → OOH* + OH^-^ | Eqn. S3 |
| --- | --- |
| OOH* + e^-^ → O* + OH^-^ | Eqn. S4 |
| O*+ H_2_O(l) + e^-^ → OH* + OH^-^ | Eqn. S5 |
| OH* + e^-^ → OH^-^ | Eqn. S6 |

Additionally, the two-electron reaction ORR pathway also exists, and both pathways are analyzed in this work. The two-electron reaction pathway is given:

| O_2_* + H_2_O(l) + e^-^ + → OOH* + OH^-^ | Eqn. S7 |
| --- | --- |
| OOH* + H_2_O(l) + e^-^ + * → H_2_O_2_(l) + OH^-^ | Eqn. S8 |

, where * represents the active site on the surface, (*l*) and (*g*) refer to liquid and gas phases, respectively, and O^*^, OH^*,^ and OOH^*^ are adsorbed intermediates on the Mo-based catalyst. We calculated the reaction Gibbs free energies of the intermediates of O*, OOH*, and OH* on the Mo(111), MoC(111), Mo@PdN_3_O_1,_ and Mo@PdN_3_PrO_1_ to determine the potential-determining step of ORR, considering all possible metal active sites. For each step, the reaction Gibbs free energy ΔG_ads_ can be expressed by


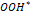


| ΔG_ads_ = ΔE_ads_ + Δ ZPE- TΔS | Eqn. S9 |
| --- | --- |

where ZPE is the zero-point energy, T is the temperature, and ΔS is the entropy change. Using

this equation (S9), we can construct a free energy diagram (FED) considering the following equations at standard conditions. The reaction Gibbs free energy of each electrochemical reaction of ORR in alkaline media can be expressed as follows.

Four-electron pathway:

| ΔG_1_ = (G_OOH*_ + μ_OH–_) – G_*_ – μ_O2_ – μ_H2O(l)_ – μ_e–_ | Eqn. S10 |
| --- | --- |
| ΔG_2-1_ = (G_O*_ + μ_OH–_) – G_OOH*_ – μ_e–_ | Eqn. S11 |
| ΔG_3_ = (G_OH*_ + μ_OH–_) – G_O*_ – μ_H2O(l)_ – μ_e–_ | Eqn. S12 |
| ΔG_4_ = (G_*_ + μ_OH–_) – G_OH*_ – μ_e–_ | Eqn. S13 |

Two-electron pathway:

| ΔG_1_ = (G_OOH*_ + μ_OH–_) – G_*_ – μ_O2_ – μ_H2O(l)_ – μ_e–_ | Eqn. S14 |
| --- | --- |
| ΔG_2-2_ = (μ_H2O2(l)_ + μ_OH–_) – G_*_ – G_OOH*_ – μ_H2O(l)_ – μ_e–_ | Eqn. S15 |

These changes in the free energy can be calculated using the chemical potential of hydroxide, electrons, liquid water, and oxygen molecules ($\mu_{\mathrm{OH}^{-}}$, $\mu_{e^{-}}$, $\mu_{H_{2}O(l)}$, and $\mu_{O_{2}}$), and the free energy of intermediate (G_OH*_, G_O*_ and G_OOH*_ on the surface *). From the calculated ΔG^ORR^ values, we can determine the critical parameter for electrocatalytic activity, which is the size of the ORR potential-determining step (G^ORR^) in the process. This is the specific reaction point with the largest ΔG in the ORR elementary reaction steps, i.e., the concluding step to achieve a downhill reaction in the free energy diagram (FED) with increasing potential:

| G^ORR^ = max [ΔG_1_, ΔG_2,_ ΔG_3_, ΔG_4_]^0^ | Eqn. S16 |
| --- | --- |

After calculating the largest ΔG, representing the bottleneck point for the ORR, we can calculate the theoretical overpotential in an alkaline media using the following equation:

| η^ORR^ = (G^ORR^/e) – xV, x = 0.402 V | Eqn. S17 |
| --- | --- |

**Table S1.** Calculated reaction free energies and overpotential (η^ORR^) for all Mo-based catalysts. ∆G_2-1_ and ∆G_2-2_ denote the second step of the 4e^-^ ORR (forming OH^-^) and the 2e^-^ ORR (forming H_2_O_2_), respectively.

| Catalysts | ∆G_1_ (eV) | ∆G_2-1_ (eV) | ∆G_2-2_ (eV) | ∆G_3_ (eV) | ∆G_4_ (eV) |
| --- | --- | --- | --- | --- | --- |
| Mo(111) | -6.29 | 2.38 | 7.08 | 0.68 | 1.62 |
| MoC(111) | -5.79 | 1.01 | 6.22 | 1.78 | 1.39 |
| Mo@Pd$N_{3}O_{1}$ | -5.90 | 0.30 | 6.58 | 1.71 | 2.28 |
| Mo@Pd$N_{3}$Pr$O_{1}$ | -5.38 | 0.08 | 6.96 | 1.68 | 2.02 |
| $O_{sur}^{*}/$Mo(111) | -1.35 | -2.01 | 2.14 | 0.32 | 1.43 |
| $O_{sur}^{*}/$MoC(111) | -1.48 | -2.32 | 2.28 | 0.78 | 1.42 |
| $O_{sur}^{*}/$Mo@Pd$N_{3}O_{1}$ | -0.06 | -0.64 | 0.73 | -0.40 | -0.50 |
| $O_{sur}^{*}/$Mo@Pd$N_{3}$Pr$O_{1}$ | -0.23 | -1.09 | 1.02 | -0.26 | -0.02 |

| **Catalyst** | **E_1/2_**  **(V_RHE_)** | **J_lim_**  **(mA cm^-2^)** | **Stability** | **Ref** |
| --- | --- | --- | --- | --- |
| **Mo-N/C SAC** | **0.88** | **-5** | **50 h, 85%** | **This work** |
| Mo-O/N-C SAC | 0.85 | -5.3 | 10 h, 94% | [1] |
| Mo1/OSG-H SAC | 0.7 | -2.78 | 8 h, 91% | [2] |
| VMoON@NC SAC | 0.861 | -6.29 | 100 h, 95.4% | [3] |
| Mo-Carbon SAC | 0.788 | -4.76 | 5.56 h, 86.4% | [4] |
| Mo SACs/N-C SAC | 0.83 | -6 | 12 h, 72% | [5] |
| SA-Mo-C-900 SAC | 0.86 | -6 | 20 h, 96.9% | [6] |
| MoC@FeNC-50 | 0.83 | -5.63 | 11.1 h, 90.5% | [7] |
| MoS2–NGO-10 | 0.72 | -4 | - | [8] |
| MoS_2_/G-500 | 0.8 | -4.5 | 45 h, 75% | [9] |
| Pd nanocubes@Mo/C | 0.84 | -5.88 | 11 h, 90.6% | [10] |
| Mo–PtFe/C–H | 0.92 | -6 | 5000 scans 94.07% | [11] |
| Pt_3_Ni_3_MoN/C | 0.95 | -6 | ADT test 30k 97.9% | [12] |
| CuPc–MoS_2_ | 0.51 | -2.36 | 2.22 h, 97.6% | [13] |
| CuMo₂ON@NG | 0.875 | -6.04 | 11.1 h, 93.1% | [14] |
| FeCoMoS@NG | 0.83 | -4.7 | 10 h, 93% | [15] |
| B,N–Mo₂C/NPNC | 0.83 | -9.5 | 2.5 h, 91% | [16] |
| MoO₃/rGO | 0.7 | -3.04 | 3 h, 92% | [17] |
| Mo₂C/NHC | 0.84 | -5 | 25 h, 77.21% | [18] |
| Mo₂C@NPC | 0.9 | -5.63 | 5.56 h, 94.8% | [19] |
| Co/MoC@N–C | 0.824 | -5 | 10 h, 91% | [20] |
| GO–Co@MoNi | 0.855 | -4.2 | 5 h, 92.7% | [21] |
|  |  |  |  |  |

**Table S2.** Benchmark comparison of recent Mo-based ORR catalysts in 0.1 M KOH electrolyte with respect to E_1/2_, J_lim_, and stability.

**References**

[1] C. Wang, D. Wang, S. Liu, P. Jiang, Z. Lin, P. Xu, K. Yang, J. Lu, H. Tong, L. Hu, *J. Catal.* **2020**, *389*, 150.

[2] C. Tang, Y. Jiao, B. Shi, J. N. Liu, Z. Xie, X. Chen, Q. Zhang, S. Z. Qiao, *Angew. Chem.* **2020**, *132*, 9256.

[3] J. Balamurugan, P. M. Austeria, J. B. Kim, E. S. Jeong, H. H. Huang, D. H. Kim, N. Koratkar, S. O. Kim, *Adv. Mater.* **2023**, *35*, 2302625.

[4] Y. Zhao, H. Wu, Y. Wang, L. Liu, W. Qin, S. Liu, J. Liu, Y. Qin, D. Zhang, A. Chu, *Energy Storage Mater.* **2022**, *50*, 186.

[5] Z. Kou, W. Zang, Y. Ma, Z. Pan, S. Mu, X. Gao, B. Tang, M. Xiong, X. Zhao, A. K. Cheetham, *Nano Energy* **2020**, *67*, 104288.

[6] F.-X. Ma, G. Zhang, M. Wang, X. Liang, F. Lyu, X. Xiao, P. Wang, L. Zhen, J. Lu, L. Zheng, *J. Colloid Interface Sci.* **2022**, *620*, 67.

[7] Q. Li, J. Zhao, P. Li, Z. Xu, J. Feng, B. Chen, R. Liu, *ACS nano* **2024**, 18, 21975.

[8] G. Yang, Y. Li, X. Wang, Z. Zhang, Y. Cai, L. Cui, C. Tan, H. Wang, Q. Li, *J. Alloys Compd.* **2022**, *904*, 164092.

[9] A. Arunchander, S. G. Peera, A. Sahu, *J. Power Sources* **2017**, *353*, 104.

[10] W. Yan, W. Wu, K. Wang, Z. Tang, S. Chen, *Int. J. Hydrogen Energy* **2018**, *43*, 17132.

[11] C. He, Z. Ma, Q. Wu, Y. Cai, Y. Huang, K. Liu, Y. Fan, H. Wang, Q. Li, J. Qi, *Electrochim, Acta.* **2020**, *330*, 135119.

[12] S. Feng, J. Lu, L. Luo, G. Qian, J. Chen, H. S. Abbo, S. J. Titinchi, S. Yin, *J. Energy Chem.* **2020**, *51*, 246.

[13] M. Samanta, S. Ghosh, M. Mukherjee, B. Das, C. Bose, K. K. Chattopadhyay, *Int. J. Hydrogen Energy* **2022**, *47*, 6710.

[14] J. Balamurugan, T. T. Nguyen, N. H. Kim, D. H. Kim, J. H. Lee, *Nano Energy* **2021**, *85*, 105987.

[15] S. Ramakrishnan, J. Balamurugan, M. Vinothkannan, A. R. Kim, S. Sengodan, D. J. Yoo, *Appl. Catal. B: Environ.* **2020**, *279*, 119381.

[16] C. He, Y. Cai, Z. Ma, X. Zhong, H. Wang, Q. Li, Y. Huang, *ACS Appl. Nano Mater.* **2021**, *4*, 8897.

[17] J. R. Bai, K. B. Pu, K. Zhang, Y. H. Wang, *J. Chem. Technol. Biotechnol.* **2022**, *97*, 3459.

[18] H. Zhao, S. Yang, W. Yang, C. Zhao, M. Cao, R. Cao, *ChemElectroChem* **2022**, *9*, e202200141.

[19] L. Ye, Y. Ying, D. Sun, J. Qiao, H. Huang, *Nanoscale* **2022**, *14*, 2065.

[20] H. Huang, L. Kong, M. Liu, J. He, W. Shuang, Y. Xu, X.-H. Bu, *J. Energy Chem.* **2021**, *59*, 538.

[21] M. Zhang, D. Qi, Y. Xin, X. Hu, T. Cao, Y. Jin, K. Wang, Z. Zhou, L. Yang, J. Jiang, *Mol. Catal.* **2022**, *528*, 112513.
